# Medicinal plants as biological and socio-ecological components of One Health

**DOI:** 10.3389/fphar.2026.1868266

**Published:** 2026-08-12

**Authors:** Ziyu Ng, Grace En Yu Xu, Shaine Lee, Yun Jin Kim

**Affiliations:** 1 School of Traditional Chinese Medicine, Xiamen University Malaysia, Selangor, Malaysia; 2 Department of Pharmacology and Therapeutics, College of Medicine and Health Sciences, United Arab Emirates University, Al Ain, United Arab Emirates

**Keywords:** biodiversity conservation, medicinal plants, One Health, phytochemistry, zoonotic diseases

## Abstract

Although the World Health Organization (WHO) estimates that approximately 40% of contemporary pharmaceuticals are derived from natural products and traditional knowledge sources, medicinal and aromatic plants (MAPs) remain underrepresented in global health policy frameworks. While the One Health concept emphasizes the interdependence of human, animal, and environmental health, MAPs have historically been viewed mainly as passive resources rather than as dynamic biological and ecological actors. This review synthesizes interdisciplinary evidence published from 2015 to 2025 to reframe medicinal plants across the One Health continuum. By examining their potential role in antimicrobial resistance mitigation through phytochemical synergy, their contribution to ethnoveterinary practices that may support animal health management and zoonotic risk reduction, and their value as biotic indicators of ecosystem integrity, this study highlights how MAPs may help connect biodiversity conservation with global health security. The available evidence suggests that selected medicinal plants and plant-derived compounds may contribute to antimicrobial resistance mitigation through mechanisms such as biofilm inhibition, efflux pump suppression, and synergistic interactions with conventional antimicrobials. The review also highlights the growing vulnerability of medicinal plant resources to biodiversity loss, climate changes in secondary metabolite production, and the erosion of traditional knowledge systems. Based on these findings, we propose an integrated One Health framework for medicinal plants that supports transdisciplinary policies linking conservation biology with pharmaceutical and healthcare resilience. Recognizing medicinal plants as biological and socio-ecological mediators may strengthen efforts to address antimicrobial resistance, biodiversity loss, and emerging global health challenges.

## 1 Introduction

Medicinal plants remain essential resources within global health systems; however, increasing global demand and human exploitation of nature pose growing threats to their sustainability. According to the World Health Organization (WHO) report on Traditional and Complementary Medicine in 2019, approximately 40% of contemporary pharmaceutical products originate from natural sources and traditional knowledge, including well-established medicines such as aspirin, artemisinin and childhood cancer treatments (World Health Organization, 2019). One Health is a collaborative concept that acknowledges the deep interconnections among human health, animal health and environmental health (Pitt and Gunn, 2024; World Health Organization et al., 2023). Although the One Health framework promotes holistic and integrative thinking, medicinal plants have rarely been studied as dynamic biological and ecological components within One Health-related research. Medicinal plants may serve not only as therapeutic agents for humans and animals, but also as ecological indicators through environmentally induced changes reflected in phytochemistry, while simultaneously acting as cultural and economic buffers that support livelihoods and ethnomedicine. Disruptions at any level propagate across the One Health continuum.

Although research on medicinal plants has expanded in pharmacology, ethnomedicine, ethnoveterinary medicine, and biodiversity conservation, these fields are often investigated independently. There remains limited understanding of how medicinal plants simultaneously influence and are influenced by interconnected human, animal, and environmental health systems. Furthermore, few studies have examined medicinal plants as biological and socio-ecological mediators capable of linking antimicrobial resistance management, zoonotic disease prevention, biodiversity conservation, and healthcare resilience within a unified One Health framework. This represents an important knowledge gap in current One Health research.

Therefore, the aim of this review is to examine the multifaceted roles of medicinal plants within the One Health framework by integrating evidence from human health, animal health, and environmental sciences. This review seeks to: (i) evaluate the therapeutic and antimicrobial applications of medicinal plants in human health; (ii) examine their roles in ethnoveterinary medicine and zoonotic disease management; (iii) assess the environmental and biodiversity challenges affecting medicinal plant resources; and (iv) propose an integrated One Health framework that positions medicinal plants as biological and socio-ecological mediators linking ecosystem integrity, animal health, and human well-being.

## 2 Methodology

### 2.1 Study design

This narrative review aims to critically examine the interconnections between human health, animal health, and environmental sustainability within the One Health framework, with particular emphasis on medicinal plants and their roles as biological and socio-ecological mediators. The review was conceptually grounded in the tripartite One Health definition advanced by the WHO, Food and Agriculture Organization, and World Organization for Animal Health, which recognizes the interdependence of people, animals, plants, and their shared environment.

### 2.2 Search strategy

A comprehensive literature search was conducted across multiple electronic databases, including PubMed, Scopus, and Web of Science. These databases were selected to ensure broad interdisciplinary coverage across biomedical sciences, environmental health, veterinary sciences, and sustainability research. The search was restricted to publications from January 2015 to December 2025 to reflect recent developments in One Health scholarship and sustainability discourse. The final literature search was conducted in December 2025; therefore, studies published in 2026 were not included.

Search terms were developed iteratively using Boolean operators and adapted to the search syntax of each database. The primary search strategy focused on the intersection between One Health and medicinal plants using the following search string: [(“One Health” OR “EcoHealth”) AND (“medicinal plants” OR “traditional medicine” OR “herbal medicine”)]. To ensure comprehensive coverage of topics relevant to this review, supplementary searches were also conducted using combinations of keywords: (“medicinal plants” AND ethnoveterinary), (“medicinal plants” AND “zoonotic diseases”), (“medicinal plants” AND biodiversity), (“medicinal plants” AND sustainability), (“traditional medicine” AND “One Health”). For PubMed, searches were performed within the Title/Abstract fields; For Scopus, the TITLE-ABS-KEY function was used; and for Web of Science, Topic searches were conducted. Retrieved records were exported and screened according to the predefined inclusion and exclusion criteria. Additional relevant articles were identified through citation tracking of key publications.

The literature search identified a total of 256 records across the three databases, including PubMed (n = 23), Scopus (n = 126), and Web of Science (n = 107). After removal of duplicate records and screening according to the predefined inclusion and exclusion criteria, 58 reports were assessed for eligibility. Following full-text evaluation, 26 studies were retained for the final narrative synthesis. Additional references were identified through citation tracking of key publications and incorporated where appropriate to provide conceptual, methodological, and contextual support. The final selection included studies providing empirical evidence, mechanistic insights, ecological assessments, policy perspectives, and conceptual frameworks relevant to medicinal plants and the One Health approach. The literature selection process is summarized in Supplementary Figure 1 (PRISMA 2020 flow diagram).

### 2.3 Eligibility criteria

#### 2.3.1 Inclusion criteria

Studies were included if they met the following criteria:

- Published in peer-reviewed journals and written in English.
- Published between January 2015 and December 2025.
- Examined medicinal plants, medicinal plant-derived compounds, ethnomedicine, ethnoveterinary medicine, biodiversity conservation, antimicrobial resistance, zoonotic disease management, or sustainability within a One Health context.
- Investigated interactions between at least two components of the One Health framework (human health, animal health, or environmental health).
- Reported original research findings, systematic reviews, narrative reviews, meta-analyses, policy analyses, ecological assessments, or conceptual frameworks relevant to medicinal plants and One Health.
- Provided sufficient methodological information and clearly described outcomes, observations, or conclusions relevant to the objectives of this review.

#### 2.3.2 Exclusion criteria

Studies were excluded if they:

- Focused exclusively on synthetic pharmaceuticals without reference to medicinal plants or plant-derived products.
- Were conference abstracts, editorials, commentaries, opinion articles, news reports, book reviews, or non-peer-reviewed publications.
- Did not address any component of the One Health framework or lacked relevance to human, animal, or environmental health.
- Did not provide sufficient methodological detail or scientific evidence to support their conclusions.
- Were published in languages other than English because translation resources were not available and reliable evaluation of scientific content could not be ensured.
- Focused solely on agricultural, industrial, or commercial plant applications without relevance to medicinal use, health outcomes, or biodiversity conservation.
- No geographical restrictions were applied. Studies from all countries and regions were considered eligible provided they met the predefined inclusion criteria.

#### 2.3.3 Study selection process

This study was conducted as a narrative review. Therefore, the literature search was intended to identify representative and scientifically relevant studies rather than to systematically retrieve all available publications. Articles were screened based on title, abstract, and full-text relevance to the objective of the review. Records that did not meet the predefined inclusion criteria were excluded during the screening process. The selection process was conducted iteratively to identify representative and scientifically relevant studies addressing medicinal plants within the One Health framework.

Studies were selected according to their contribution to understanding the role of medicinal plants within the One Health framework, including their applications in human health, antimicrobial resistance management, ethnoveterinary medicine, zoonotic disease prevention, biodiversity conservation, and sustainable resource management. Additional relevant references were identified through citation tracking of key publications. The final selection emphasized studies that provided substantial empirical evidence, conceptual insights, or comprehensive reviews relevant to the scope of this article.

#### 2.3.4 Selection of medicinal plant examples

The medicinal plants highlighted in this review were selected based on their frequency of reporting in peer-reviewed literature, documented traditional use across different geographical regions, and the availability of scientific evidence supporting their therapeutic, antimicrobial, ethnoveterinary, or zoonotic disease-related applications. The examples presented are intended to illustrate representative and well-studied medicinal plants relevant to the One Health framework and do not constitute an exhaustive list of all potentially useful species. Preference was given to plant species with multiple independent reports demonstrating biological activity or established traditional use in human and animal healthcare systems.

## 3 Different perspectives of One Health

### 3.1 Human health perspective

From a human health perspective, medicinal plants remain important resources within many traditional and complementary healthcare systems. Evidence from ethnomedical reports, laboratory investigations, animal studies and selected clinical studies suggests that some medicinal plants may have relevance to chronic diseases, infectious diseases and antimicrobial resistance. The WHO reports that 70% to 80% of the global population relies on medicinal plants as their primary or secondary healthcare, especially in developing countries (World Health Organization, 2019; World Health Organization et al., 2023).

#### 3.1.1 Therapeutic uses of medicinal plants

The medicinal plants discussed in this section were selected as representative examples because of their widespread traditional use and substantial documentation in the scientific literature. Medicinal plants are used for a wide range of human health conditions, including malaria, which remains a major public health threat in Africa. In Ethiopia, *Allium sativum* L. (Amaryllidaceae) has been reported in traditional medicine for malaria treatment. In addition, plants such as *Alchornea cordifolia* (Schumach. and Thonn.) Müll.Arg. (Phyllanthaceae), *Cryptolepis sanguinolenta* (Lindl.) Schltr. (Apocynaceae), and *Zanthoxylum chalybeum* Engl. (Rutaceae) have been documented for antimalarial activity (Tajbakhsh et al., 2021). *Salix alba* L. (Salicaceae), commonly known as willow bark, has historically been used for pain and inflammation, while *Rubia cordifolia* L. (Rubiaceae) has been investigated for anti-inflammatory properties (Misganaw et al., 2025; Kumar and Sharma, 2025; Sen and Samanta, 2014). Several Rutaceae species have been reported to contain terpenoids, flavonoids, alkaloids, and essential oils. Certain compounds isolated from these species have demonstrated bronchodilatory, anti-inflammatory, or antioxidant activities under experimental conditions (Kumar and Sharma, 2025; Siddique et al., 2022). In Ethiopia, medicinal plants such as *Dodonaea viscosa* subsp. *Angustifolia* (L.f.) J.G.West (Sapindaceae), *Clematis simensis* Fresen. (Ranunculaceae), and *Capparis tomentosa* Lam (Capparaceae). Have been reported for wound healing and skin infections, and *Salvia rosmarinus* Spenn. (Lamiaceae). Have been used for headache and migraine. For pregnancy and women’s health, *Combretum micranthum* G. Don (Combretaceae) has been used in Mali for oedema. *Parkia biglobosa* (Jacq.) R. Br. ex G. Don (Fabaceae) for urinary tract infections, *Linum usitatissimum* L. (Linaceae), commonly known as flaxseed in Ethiopia to induce or shorten labor, and *Saraca asoca* (Roxb.) Willd. (Fabaceae) in Ayurvedic medicine for heavy menstrual bleeding. Other plants, such as *Wrightia tinctoria* (Roxb.) R. Br. (Apocynaceae), have also been reported in postnatal care (Kumar and Sharma, 2025; Nergard et al., 2015; Ahmed et al., 2021; Zawude et al., 2022).

These examples show that medicinal plants are frequently used for infectious diseases, inflammatory disorders, reproductive health conditions, and wound management. Although the reported species originate from diverse geographical regions and cultural traditions, many contain common phytochemical classes, including flavonoids, alkaloids, terpenoids, and phenolic compounds. This overlap suggests that related biological pathways may contribute to their therapeutic effects, although these effects remain species- and compound-specific.

The evidence supporting the medicinal applications discussed in this section varies substantially in quality and scope. Many uses, particularly those related to malaria, reproductive health, and wound management, are primarily documented through ethnomedical reports and traditional healthcare practices. Other applications have been investigated through laboratory-based studies that evaluate antimicrobial, anti-inflammatory, or antioxidant activities under controlled conditions. A smaller number of medicinal plants have been examined in animal models, while robust clinical evidence remains limited for many species. Although traditional and preclinical findings provide valuable insights, further clinical investigations are required to establish safety, efficacy, dosage, and long-term therapeutic outcomes.

#### 3.1.2 Effects of medicinal plants on chronic diseases

Medicinal plants have been used in the management of chronic conditions, including cancer, obesity, metabolic disorders, cardiovascular diseases, inflammatory disorders, and pain. Several medicinal plants have been investigated for potential antineoplastic effects in preclinical or selected clinical studies; examples include *Curcuma longa* L. (Zingiberaceae; turmeric), *Camellia sinensis* (L.) Kuntze (Theaceae; green tea), *Viscum album* L. (Santalaceae; mistletoe) and *Panax ginseng* C.A.Mey. (Araliaceae; ginseng). Compounds from seeds of *Celastrus paniculatus* Willd. (Celastraceae; black oil plant) have been reported to induce apoptosis in breast cancer cells (Kumar and Sharma, 2025). Traditional healers in Ethiopia also report the use of plants such as *Phytolacca dodecandra* L'Hér. (Phytolaccaceae; endod) for tumors (Misganaw et al., 2025).

Plants such as green tea (*C. sinensis* (L.) Kuntze; Theaceae), ginger (*Zingiber officinale* Roscoe; Zingiberaceae), cocoa (*Theobroma cacao* L.; Malvaceae), and cinnamon (*Cinnamomum* spp.) have been investigated for their potential role in obesity management. Their bioactive constituents, including epigallocatechin gallate (EGCG) from *C. sinensis* (L.) Kuntze (Theaceae; green tea) and cinnamaldehyde from *Cinnamomum* spp. (Lauraceae; cinnamon), have been reported to influence pathways associated with energy expenditure, appetite regulation, and lipid metabolism in experimental and selected clinical studies (Alshammaa and AlShammaa, 2024). Traditional remedies for diabetes include *Gymnema sylvestre* (Retz.) R. Br. ex Sm. (Apocynaceae), *Azadirachta indica* A. Juss. (Meliaceae; neem), and *Apteranthes tuberculata* (N.E.Br.) Meve and Liede (Apocynaceae) (Siddique et al., 2022; Zawude et al., 2022).

Plants such as *Hippophae rhamnoides* L. (Elaeagnaceae; sea buckthorn), *Allium sativum* L. (Amaryllidaceae; garlic), and *Silybum marianum* (L.) Gaertn. (Asteraceae; milk thistle) have been used to support blood pressure or cholesterol management. Reserpine, a conventional antihypertensive and psychotic agent, is derived from the roots of *Rauvolfia serpentina* (L.) Benth. ex Kurz (Apocynaceae) and has historically been used to treat mild to moderate hypertension (Kumar and Sharma, 2025; Siddique et al., 2022; Zawude et al., 2022; Alanazi et al., 2025).

Species belonging to the Rutaceae family (e.g., *Citrus aurantium* L. (Rutaceae) and *Zanthoxylum chalybeum* Engl. (Rutaceae).), together with *Andrographis paniculata* (Burm.f.) Nees (Acanthaceae), have demonstrated anti-inflammatory activity associated with diverse phytochemical constituents, including flavonoids, alkaloids, and terpenoids. Plants such as *Trichilia emetica* Vahl (Meliaceae), *Alangium salviifolium* (L.f.) Wangerin (Cornaceae), and *Ocimum lamiifolium* Hochst. ex Benth. (Lamiaceae) have traditionally been used for pain relief. *Hemidesmus indicus* (L.) R. Br. ex Schult. (Apocynaceae) has traditionally been used in Ayurvedic medicine to support immune function (Kumar and Sharma, 2025; Nergard et al., 2015; Ahmed et al., 2021; Zawude et al., 2022; Kuo, 2015).

A frequently proposed advantage of medicinal plants in chronic disease management is their potential to influence multiple biological pathways. Because medicinal plants often contain several bioactive constituents, they may affect antioxidant, inflammatory, metabolic, or immunomodulatory pathways simultaneously. This multi-component activity may partly explain their widespread use in complex chronic diseases such as cancer, diabetes, obesity, and cardiovascular disorders. However, the quality of evidence supporting these applications differs considerably among species and disease categories.

While certain plants such as *Curcuma longa* L. (Zingiberaceae; turmeric) and *C. sinensis* (L.) Kuntze (Theaceae; green tea) have been investigated through laboratory studies, animal models, and selected clinical trials, many other reported applications remain supported primarily by ethnomedical knowledge or preclinical evidence. Therefore, caution should be exercised when extrapolating laboratory findings to clinical practice, and additional well-designed clinical studies are needed to confirm therapeutic effectiveness and safety.

#### 3.1.3 Medicinal plants and antimicrobial resistance

Numerous medicinal plants have demonstrated antimicrobial activity against bacteria, fungi, viruses, and parasites under experimental conditions (Zouine et al., 2024). These activities have been attributed to diverse classes of plant-derived secondary metabolites, including specific flavonoids, terpenoids, alkaloids, phenolic acids, tannins, glycosides, and saponins (Gorlenko et al., 2020). However, antimicrobial efficacy varies considerably according to the chemical structure of individual compounds, extract composition, target microorganisms, and experimental conditions. Rather than acting through a single mechanism, different phytochemicals have been reported to interfere with multiple microbial processes, including membrane integrity, energy metabolism, biofilm formation, quorum sensing, virulence factor expression, and antimicrobial resistance mechanisms. The mechanisms discussed below should therefore be interpreted as compound-specific rather than representative of entire phytochemical classes (Kumar et al., 2023; De Rossi et al., 2025).

##### 3.1.3.1 Cell wall and membrane disruption and lysis

Certain terpenoids, flavonoids, and alkaloids have been reported to disrupt microbial membrane integrity through mechanisms depending on their chemical structures. Proposed mechanisms include interactions with phospholipid bilayers, increased membrane permeability, dissipation of transmembrane ion gradients, and impairment of energy metabolism. These activities are highly structure-dependent and differ substantially among individual compounds and microbial species (Luo et al., 2025; Khanam et al., 2025). For example, some flavonoids, including pinostrobin isolated from *Boesenbergia rotunda* (L.) Mansf. (Zingiberaceae; fingerroot) and other prenylated flavonoids, have been reported to increase membrane permeability in *Enterococcus faecalis*, *Staphylococcus aureus*, *Escherichia coli*, and *Pseudomonas aeruginosa*. Glabridin, a major isoflavan isolated from *Glycyrrhiza glabra* L. (Fabaceae; licorice), has also been reported to disrupt fungal cell wall integrity, suggesting a possible mechanism for antifungal action (Yang et al., 2021).

In addition, cationic antimicrobial peptides from plants can interact with negatively charged microbial cell membranes, leading to pore formation and altered membrane permeability (So et al., 2025). However, most evidence supporting membrane disruption originates from *in vitro* susceptibility assays, and comparatively few studies have confirmed these mechanisms in animal infection models or clinical settings.

##### 3.1.3.2 Inhibition of biofilm formation

Several plant-derived compounds have demonstrated antibiofilm activity under experimental conditions. Among them, specific flavonoids, including galangin and EGCG (Rabin et al., 2015), have been reported to interfere with biofilm development by modulating quorum-sensing pathways and reducing the expression of genes associated with adhesion and virulence. However, reported activities differ among bacterial species, compound structures, concentrations, and experimental models, limiting direct comparison across studies (Hasnat et al., 2024).

Specific flavonoids may interfere with quorum-sensing systems by reducing the production, release, or recognition of signaling molecules such as N-acyl homoserine lactones and autoinducing peptides, depending on their chemical structures. Through disruption of these signaling pathways, some flavonoids may downregulate genes associated with biofilm formation, virulence factor production, adhesion, and microbial communication (Lim et al., 2019). For example, phloretin has been reported to inhibit fimbriae formation in *E. coli* by downregulating curli genes (csg A, csgB) and toxin genes (hemolysin E and Shiga toxin 2), thereby impeding biofilm development (Qiu et al., 2025; Lee et al., 2011). Overall, most antibiofilm findings are based on laboratory biofilm models, and their clinical relevance remains to be established.

##### 3.1.3.3 Inhibition of efflux pumps

Efflux pumps are a major mechanism of antibiotic resistance because they actively expel antibiotics from the bacterial cells (Kumawat et al., 2023). Several plant-derived compounds have demonstrated efflux pump inhibitory activity. For example, the isoquinoline alkaloid berberine has been reported to inhibit the NorA multidrug efflux pump in methicillin-resistant *Staphylococcus aureus* (MRSA), particularly when combined with the efflux pump inhibitor 5′-methoxyhydnocarpin (5′-MHC). Other phytochemicals, including certain phenolic compounds and terpenoids, have also been investigated. However, the effectiveness of efflux pump inhibition varies substantially among compounds and bacterial species (Yang et al., 2025). A seminal study showed that 5′-MHC enhances the antibacterial activity of berberine against *S. aureus* by inhibiting bacterial efflux mechanisms, thereby increasing intracellular berberine accumulation (Stermitz et al., 2000). Terpenes such as carvacrol, a major constituent of *Origanum vulgare* L. (Lamiaceae; oregano), and *Thymus vulgaris* L. (Lamiaceae; thyme), have also been investigated as potential efflux pump inhibitors in *S. aureus* (Dias et al., 2022). Although efflux pump inhibition represents a promising strategy for antimicrobial potentiation, evidence remains largely restricted to *in vitro* investigations.

##### 3.1.3.4 Inhibition of microbial growth, replication, and metabolism

Specific compounds, such as emodin, an anthraquinone derivative isolated from *Rheum palmatum* L. (Polygonaceae; Chinese rhubarb), and baicalein, a flavonoid derived from *Scutellaria baicalensis* Georgi (Lamiaceae; Baikal skullcap), have been reported to affect microbial growth, replication, and metabolism (Zhu et al., 2025). Proposed mechanisms include interference with cell wall construction, microbial DNA replication, energy synthesis, and reactive oxygen species (ROS) production. Emodin has been reported to prevent the transfer of resistance genes in *S. aureus* (Min et al., 2017), Baicalein has also been reported to suppress SOS-response genes (RecA, LexA, and SACOL1400), thereby reducing the emergence of rifampin-resistant mutations in *S. aureus* (Peng et al., 2011). Phenolic compounds such as carnosic and rosmarinic acids have also been reported to affect cmeB, cmeF, and cmeR genes in *Campylobacter jejuni* (Klančnik et al., 2012).

##### 3.1.3.5 Attenuation of bacterial virulence and toxin production

Some plant-derived compounds have been reported to attenuate bacterial virulence by reducing toxin production or interfering with quorum-sensing pathways. For example, phloretin reduces the expression of toxin genes, including hemolysin and Shiga toxin 2, in *E. coli* (Geohagen et al., 2018). Resveratrol, a polyphenol found in *Vitis vinifera* L. (Vitaceae; grapevine) and curcumin, the principal curcuminoid of *Curcuma longa* L. (Zingiberaceae; turmeric) have been reported to reduce toxin-related gene expression in MRSA. Other plant polyphenols have also been investigated for inhibition of staphylococcal alpha-toxin (El-Mahdy et al., 2024).

##### 3.1.3.6 Modulation of host immune responses

Certain plant-derived polysaccharides and specific saponins isolated from selected medicinal plants have demonstrated immunomodulatory activities that may support host defense against MDR microorganisms. These compounds have been reported to activate immune cells, including macrophages and neutrophils, thereby enhancing phagocytic activity (Nam et al., 2024). However, immunomodulatory effects remain compound-specific and require further validation in relevant infection models.

##### 3.1.3.7 Synergistic effects with conventional antibiotics

Evidence from predominantly *in vitro* studies suggests that selected medicinal plants extracts or plant-derived compounds may demonstrate synergistic effects when combined with conventional antibiotics, thereby enhancing activity against MDR pathogens. Such interactions may potentially reduce the required dose of conventional drugs and delay the development of resistance, although this remains to be validated *in vivo*. For instance, several *in vitro* studies have reported synergistic effects between baicalein and tetracycline or β-lactams against MRSA (Fujita et al., 2005). Flavonoids isolated from smaller galangal have shown synergistic activity with amoxicillin against amoxicillin-resistant *E. coli* (Eumkeb et al., 2012). Garlic extract combined with vinegar has demonstrated increased antibiotic activity (Cocom et al., 2025). Punicalagin, a pomegranate polyphenol, has been reported to potentiate antibiotic activity against three MDR pathogens of the Enterobacteriaceae family (Ki et al., 2024). Supplementary Table 1 summarizes representative medicinal plants, major bioactive compounds, and reported therapeutic applications in human health. Further studies are needed to evaluate the consistency of active compounds, potential adverse effects, pharmacokinetics, and clinical relevance of medicinal plant-antibiotic combinations.

The evidence supporting the antimicrobial activities described in this section is derived predominantly from *in vitro* investigations, including antimicrobial susceptibility testing, biofilm inhibition assays, and mechanistic studies of phytochemical activity. Comparatively fewer studies have evaluated medicinal plant compounds in animal models, and clinical evidence remains limited for most species. As a result, many promising antimicrobial findings should be considered preliminary until validated through well-designed *in vivo* and clinical studies.

From a One Health perspective, the antimicrobial properties of medicinal plants may have implications beyond individual therapeutic applications. By reducing alternative or complementary mechanisms against multidrug-resistant pathogens, selected medicinal plants and plant-derived compounds may contribute to antimicrobial resistance mitigation across human, animal, and environmental settings. Nevertheless, most evidence currently originates from *in vitro* studies, while clinical and field-based validation remains limited. Future research should therefore prioritize translational studies that evaluate efficacy, safety, dosage standardization, pharmacokinetics, and long-term ecological impacts.

Although numerous studies have reported antimicrobial activity of medicinal plant extracts, most available evidence originates from *in vitro* investigations. Comparatively fewer studies have evaluated standardized extracts in animal models or clinical settings. In addition, variation in phytochemical composition, extraction methods, dosage, and experimental protocols limits comparison across studies and reduces reproducibility.

### 3.2 Animal health perspective

Animal health is a central component of the One Health concept because animal diseases, antimicrobial use, livestock productivity, and zoonotic transmission directly influence human health and environmental sustainability (Lymbery, 2025). Medicinal plants are relevant to this interface through their roles in ethnoveterinary medicine, livestock health management, and potential complementary use in zoonotic disease control. Medicinal plants may contribute to addressing selected animal health challenges through ethnoveterinary medicine and complementary animal healthcare. Ethnoveterinary use of medicinal plants reflects accumulated empirical knowledge transmitted across generations in local and indigenous communities.

Most evidence supporting ethnoveterinary application is derived from traditional knowledge and small-scale experimental studies. Well-controlled livestock trials remain limited, making it difficult to establish standardized dosage recommendations, long-term safety profiles, and economic feasibility under commercial production systems.

#### 3.2.1 Zoonotic disease prevention

Zoonotic diseases are infectious diseases that are naturally transmitted between animals and humans. COVID-19 is one prominent example of a disease that has been discussed within the context of zoonotic emergence (Haider et al., 2020). Diseases caused by pathogens such as Ebola virus and Nipah virus are also commonly considered zoonotic or associated with animal reservoirs (González-Barrio, 2022; Haider et al., 2020). Zoonotic diseases can be classified according to pathogen types, including viruses, bacteria, parasites, fungi, and prions, or according to their maintenance cycle, such as orthozoonoses, cyclozoonoses, metazoonoses and saprozoonoses (Waltner-Toews, 2017; Ghanbari et al., 2020).

Zoonotic diseases can affect human health, animal health, ecosystems, and economies through treatment cost, livestock losses, and disruption of sectors such as tourism and more (Carugati et al., 2019; Natterson-Horowitz et al., 2022). One Health approaches to zoonotic disease control integrate expertise from veterinarians, ecologists, physicians, public health officials, and policymakers to prevention and surveillance strategies (Rahman et al., 2020; Rodriguez-Morales et al., 2021). The strategies include integrated surveillance of pathogens in the animal populations, which may provide early warning of diseases outbreaks (Cunningham et al., 2017). Addressing upstream drivers, such as environmental degradation and agricultural intensification, may also help reduce pathogen transmission risks (Hulme, 2020; Davis and Sharp, 2020).

Several medicinal plants have demonstrated biological activities that may contribute to the management of selected zoonotic pathogens. For example, *Peganum harmala* L. (Nitrariaceae; Syrian rue) has been investigated against protozoan parasites of veterinary importance, including *Theileria spp*., suggesting potential applications in ethnoveterinary medicine (Lokhande et al., 2025). Compounds or extracts from selected medicinal plants have also been investigated against pathogens associated with zoonotic disease, including *Leishmania* spp., *Toxoplasma gondii*, and several bacterial species. Similarly, *Allium sativum* L. (Amaryllidaceae) contains sulfur-containing compounds such as allicin, which have shown inhibitory effects against selected bacterial and parasitic pathogens (Thakur et al., 2024). *Punica granatum* L. (Lythraceae; pomegranate) and *Zingiber officinale* Roscoe (Zingiberaceae; ginger) have also demonstrated antiparasitic and immunomodulatory properties that may support host defense against gastrointestinal parasites, including *Giardia duodenalis* and *Haemonchus contortus* (Attia et al., 2025).

The potential contribution of medicinal plants to zoonotic disease management may involve multiple mechanisms, including direct antimicrobial activity, inhibition of pathogen growth and replication, modulation of host immune responses, reduction of inflammation, and alteration of gut microbial balance.

In livestock systems, these properties may contribute to improved animal health and potentially reduce pathogen transmission risks. However, most evidence currently originates from laboratory studies and animal models, while field-based and clinical investigations remain limited. Therefore, medicinal plants should be viewed as potential complementary tools within integrated zoonotic disease management strategies rather than as standalone preventive measures.

#### 3.2.2 Ethnoveterinary medicine

The plant species highlighted in this section were chosen based on their frequent citation in ethnoveterinary studies and their documented applications in animal health management across different regions. Ethnoveterinary medicine refers to the knowledge, practices, and skills used by local and indigenous communities to prevent and treat animal diseases (de Farias Gonçalves et al., 2025). It is important because it may support rural economies, preserve cultural heritage, and provide accessible healthcare options for livestock in resource-limited settings. In some rural areas, livestock farming is the primary source of income, and ethnoveterinary medicine may provide a lower-cost alternative when conventional veterinary medicines are expensive or inaccessible (Rafique Khan et al., 2021). Ethnoveterinary knowledge also reflects the close relationship among people, animals, and local biodiversity (Khan et al., 2025; Ndou et al., 2023). In remote areas where conventional pharmaceuticals are limited, communities may rely on locally available medicinal plants to manage animal health conditions (Kebede et al., 2018).

Beyond their cultural and economic importance, ethnoveterinary medicinal plants have attracted increasing scientific interest because of their reported biological activities. Experimental studies have described antimicrobial, antiparasitic, anti-inflammatory, antioxidant, and immunomodulatory effects for some species used in traditional animal healthcare. These activities are frequently associated with phytochemicals such as flavonoids, alkaloids, terpenoids, tannins, and saponins, which may contribute to pathogen control, immune function, and animal productivity. However, the strength of evidence varies considerably among species, and many applications remain supported primarily by traditional knowledge and small-scale experimental studies rather than large veterinary field trials.

##### 3.2.2.1 Ethnoveterinary practices and remedies

###### 3.2.2.1.1 Plant-based remedies

Most remedies used in ethnoveterinary medicine are prepared from plants that grow locally. Plant parts used for treatment vary by location and cultural practice, although underground parts such as roots, rhizomes and tubers are often reported. Preparations commonly include pastes, decoctions, and infusions, which may be administered orally or applied topically (Mandal and Rahaman, 2022).

###### 3.2.2.1.2 Polyherbal formulations

Many ethnoveterinary remedies are polyherbal formulations, meaning that they combine multiple plants with the aim of enhancing efficacy or tolerability. Commonly used medicinal plants such as black pepper, turmeric and ginger are sometimes used as additives or bioenhancers to improve the bioavailability or absorption of active compounds from other herbs (Yurdakok-Dikmen et al., 2018).

###### 3.2.2.1.3 Other uses of medicinal plants in animal health

####### 3.2.2.1.3.1 Growth promoters and feed additives

Medicinal plants have been explored as potential alternative or complementary strategies to reduce antibiotic use in animal production systems (Kuralkar and Kuralkar, 2021). Some plant-based feed additives may improve selected performance parameters by stimulating appetite, increasing feed intake, enhancing nutrient digestibility, or modulating gut microbiota. For example, supplementation with thyme, rosemary, peppermint, garlic, or turmeric has been reported to improve selected growth or health-related parameters in poultry studies, although outcomes vary according to formulation, dosage, and production conditions (Dardouri et al., 2025). Turmeric, ginger and garlic have also been investigated for their potential to improve gut morphology and reduce harmful bacterial loads in pigs. Plants from the Polygonaceous family, such as buckwheat, have been investigated for effects on milk quality and methane or ammonia formation in ruminants, while *Rumex obtusifolius* L. (Polygonaceae; broad-leaved dock), has been reported for potential use in reducing bloat in cows (Türk et al., 2025).

####### 3.2.2.1.3.2 Animal production

Several studies have reported that certain medicinal plants may support reproductive health in animals. Reproductive disorders such as anoestrus, retained placenta, and infertility are among the conditions addressed in ethnoveterinary practice. *Asparagus racemosus* Willd. (Asparagaceae; shatavari), *Withania somnifera* (L.). Dunal (Solanaceae; ashwagandha), and *Tinospora cordifolia* (Willd.) Hook. f. and Thomson (Menispermaceae; guduchi) have been reported in ethnoveterinary or experimental contexts for reproductive health support, but robust controlled livestock trials remain limited (Kuralkar and Kuralkar, 2021).

####### 3.2.2.1.3.3 Antimicrobial and antiparasitic properties in animals

Plants from the *Polygonum* and *Rumex* genera (Polygonaceae) have been reported to show antibacterial and antifungal properties (Türk et al., 2025). Experimental studies in broiler chickens have reported that supplementation with *Thymus vulgaris* L. (Lamiaceae; thyme), *Salvia rosmarinus* Spenn. (Lamiaceae) and *Smilax glabra* Roxb. (Smilacaceae; sarsaparilla). May reduce intestinal *E. coli* while increasing beneficial *Lactobacillus* species, thereby supporting gut health and feed efficiency (McMurray et al., 2022).

Plants from the Fabaceae family are often reported for antiparasitic use. In addition, *Allium sativum* L. (Amaryllidaceae), *Zingiber officinale* Roscoe (Zingiberaceae; ginger), and *Punica granatum* L. (Lythraceae; pomegranate) have shown potential activity against gastrointestinal parasites such as *Haemonchus contortus*, *Giardia duodenalis*, and *Eimeria* spp. (Ranasinghe et al., 2023). *Bistorta officinalis* Delarbre (Polygonaceae; bistort) has also been reported to show activity against coccidial oocysts (Türk et al., 2025).

####### 3.2.2.1.3.4 Immunomodulators

Some herbs may modulate immune responses in animal and thereby support resistance to infection. *Withania somnifera* (L.) Dunal (Solanaceae; ashwagandha), *Azadirachta indica* A. Juss. (Meliaceae; neem), and *Tinospora cordifolia* (Willd.) Hook. f. and Thomson (Menispermaceae; guduchi) have been reported in poultry-related studies or traditional practice for immune support (Dhama et al., 2015).

###### 3.2.2.1.4 Bioactive compounds and mechanism of action

####### 3.2.2.1.4.1 Polyphenols (tannins and flavonoids)

Representative flavonoids include quercetin, EGCG, galangin, and baicalein, whereas condensed tannins are among the tannins most frequently investigated in ruminant nutrition. Experimental studies suggest that these compounds may exert antioxidant, antibiofilm, anti-inflammatory, and antiparasitic activities through modulation of oxidative stress pathways, quorum-sensing systems, and rumen microbial fermentation (Dardouri et al., 2025). However, these biological activities vary according to tannin type, flavonoid structure, concentration, animal species, and experimental conditions. Further validation under practical livestock conditions and well-controlled field studies is required.

####### 3.2.2.1.4.2 Alkaloids

Representative alkaloids include berberine, quinine, and harmine. Berberine has been reported to inhibit bacterial multidrug efflux pumps, whereas quinine and harmine have demonstrated antiparasitic activities against selected protozoan and helminth parasites under experimental conditions. However, these biological activities depend strongly on the chemical structures of individual alkaloids, target parasite species, dosage, and experimental conditions. Therefore, these pharmacological effects should not be generalized across the entire alkaloid class. Some alkaloids have also been reported antimicrobial and anti-inflammatory activities, although further validation is required before broad veterinary application can be recommended (Ranasinghe et al., 2023).

####### 3.2.2.1.4.3 Saponins

Saponins comprise a structurally diverse class of glycosides rather than a single compound. Representative saponins include tea saponins, Quillaja saponins, and Yucca-derived steroidal saponins. Experimental studies have investigated these compounds for their potential to reduce methane production through modulation of rumen protozoal populations and microbial fermentation. Certain saponins have also demonstrated antiprotozoal, antifungal, and molluscicidal activities under experimental conditions (Kuralkar and Kuralkar, 2021). However, these biological activities vary according to saponin structure, plant source, dosage, diet composition, rumen microbial ecology, and experimental conditions, and consistent *in vivo* evidence remains limited.

####### 3.2.2.1.4.4 Terpenoids and essential oils

Representative terpenoids and essential oil constituents, including thymol, carvacrol, eugenol, and cinnamaldehyde, have demonstrated antimicrobial, anti-inflammatory, and antioxidant activities in experimental studies (Kuralkar and Kuralkar, 2021; Dardouri et al., 2025). Proposed mechanisms include disruption of microbial membrane integrity, modulation of inflammatory signaling pathways, and effects on digestive enzyme secretion. These biological activities differ substantially among individual compounds and experimental models, and evidence supporting their effectiveness under commercial livestock conditions remains limited. Supplementary Table 2 summarizes representative medicinal plants, major bioactive compounds, and reported therapeutic applications in animal health.

Overall, evidence supporting the biological activities of phytochemical classes in ethnoveterinary medicine remains heterogeneous. Many reported mechanisms are derived from *in vitro* investigations or controlled animal experiments using purified compounds or standardized extracts, whereas comparatively few studies have evaluated naturally occurring phytochemical mixtures under practical livestock conditions. Furthermore, considerable variation in phytochemical composition among plant species, cultivation environments, extraction methods, and dosage regimens limits direct comparison across studies. Future research should therefore prioritize standardized phytochemical characterization, dose-response analyses, and well-designed veterinary field trials to improve the translational value of ethnoveterinary research.

### 3.3 Environmental and biodiversity perspective

Within the One Health framework, which recognizes the interdependence of human, animal, and environmental wellbeing, medicinal and aromatic plants (MAPs) occupy a critical intersection between nature and human health. The conservation of MAPs is closely tied to the broader challenge of maintaining ecological balance and global biodiversity. However, the growing environmental crisis of the Anthropocene has placed unprecedented stress on these interconnected systems, threatening biodiversity from genetic to ecosystem levels (Damiani et al., 2023; Theodoridis et al., 2023; Shaw et al., 2025).

Biodiversity loss today is driven primarily by climate change, pollution, land-use transformation, overexploitation of natural resources, and the spread of invasive species (Zhu et al., 2024). These direct pressures are reinforced by indirect socio-economic forces, including unsustainable production and consumption patterns, population growth, and urbanization. As a result, an estimated 35%–50% of the Earth’s terrestrial surface has been modified by human activity (Kursar et al., 2008). Expanding agriculture, mining, deforestation, and infrastructure development continue to fragment natural habitats, pushing many wild medicinal plant species toward decline or extinction (Kursar et al., 2008; Asigbaase et al., 2023; Gitima et al., 2025). The need to balance crop productivity with ecosystem health has therefore become a pressing concern for both food security and environmental sustainability.

Quantitative evidence further highlights the magnitude of biodiversity loss and its implications for medicinal plant conservation. The Intergovernmental Science-Policy Platform on Biodiversity and Ecosystem Services (IPBES) estimates that approximately one million plant and animal species are currently threatened with extinction, while the abundance of native species in many terrestrial ecosystems has declined by at least 20% since 1900 (Tollefson, 2019). According to the IUCN Red List, numerous medicinal and aromatic plant species are facing increasing extinction risks due to habitat loss, overharvesting, climate change, and land-use transformation. A recent global assessment of medicinal plant species reported that among 3,227 medicinal plants documented in trade, 954 species had global conservation assessments, of which 82 were classified as threatened, while 688 species were identified as threatened in at least one national conservation assessment (Brinckmann et al., 2022).

These trends raise concerns regarding the long-term availability of medicinal resources and the sustainability of plant-based healthcare systems. High-value medicinal plants such as *Dactylorhiza hatagirea* (D.Don) Soó (Orchidaceae), *Neopicrorhiza scrophulariiflora* (Pennell) D.Y.Hong (Plantaginaceae), and *Nardostachys jatamansi* (D.Don) DC. (Caprifoliaceae) provide examples of species experiencing population declines due to overexploitation and environmental pressures (Krattenmacher et al., 2023). The continued loss of medicinal plant diversity may reduce the availability of bioactive compounds for future drug discovery while simultaneously undermining traditional healthcare systems that depend upon these resources.

Climate change intensifies this crisis by reshaping ecosystems and amplifying the vulnerability of MAPs. Climate change may also contribute to a phenomenon described as climate-induced phytochemical variation, referring to the alteration in biologically active secondary metabolites in medicinal plants because of environmental stress (Isah, 2019). For example, drought stress and increasing temperatures have been shown to alter the concentrations of phenolic compounds and essential oils in several medicinal plant species, including *Hypericum perforatum* L. (Hypericaceae), *Salvia officinalis* L. (Lamiaceae), and *Thymus vulgaris* L. (Lamiaceae), potentially affecting their pharmacological efficacy and commercial value (Tan and Gören, 2024). Secondary metabolites, including flavonoids, phenolic acids, terpenoids, alkaloids, and glycosides, are synthesized through complex metabolic pathways such as the phenylpropanoid, flavonoid, terpenoid, and alkaloid biosynthetic pathways (Yao et al., 2026). These compounds contribute to the antimicrobial, antioxidant, anti-inflammatory, and therapeutic properties of medicinal plants. Environmental stressors associated with climate change, including elevated temperatures, prolonged drought, altered precipitation patterns, increased atmospheric CO_2_ concentrations, and soil degradation, can disrupt these metabolic pathways by altering gene expression, enzyme activity, nutrient availability, and carbon allocation within plants (Zandalinas et al., 2022).

Medicinal plants may exhibit changes in both the quantity and composition of bioactive compounds, potentially reducing their therapeutic efficacy and consistency. This climate-related metabolic alteration may therefore compromise the medicinal value of plant resources while simultaneously affecting the healthcare systems and communities that depend on them.

Shifts in temperature, precipitation, and drought frequency directly affect the growth, reproduction, and chemical composition of medicinal plants (Gaur et al., 2024). Moreover, flowering cycles disrupt pollination and reduce the regeneration of key species, while invasive plants favored by changing climatic conditions outcompete native flora, disturbing ecological balance and diminishing biodiversity. These factors threaten the availability of medicinally valuable plants that form the foundation of traditional and modern healthcare systems worldwide.

Overexploitation remains another major challenge. Many MAPs are harvested destructively, especially those whose roots, bark, or whole plants are used in traditional medicine (Asigbaase et al., 2023). Such unsustainable collection practices endanger slow-growing and habitat-specific species, leading to local depletion and even extinction. Alongside habitat loss, these pressures result in genetic erosion, the gradual loss of genetic diversity that underpins resilience and adaptive capacity in plant populations (Shaw et al., 2025; Salgotra and Chauhan, 2023). The decline of genetic variation across species is thus both a biological and cultural loss, affecting ecological stability and the continuity of ethnobotanical traditions.

Another important but often overlooked threat to medicinal plant conservation is the loss of traditional knowledge. Indigenous peoples and local communities have accumulated extensive knowledge regarding the identification, harvesting, cultivation, preparation, and therapeutic use of medicinal plants over generations. However, rapid urbanization, modernization, migration, changing lifestyles, and reduced intergenerational knowledge transfer have contributed to the erosion of this valuable cultural heritage (Alum, 2024). The loss of traditional knowledge not only reduced the effective utilization of medicinal plants but may also lead to the abandonment of sustainable harvesting and management practices that have historically supported biodiversity conservation. The disappearance of ethnobotanical knowledge represents both a cultural and ecological loss, potentially undermining future efforts to conserve medicinal plant resources and develop novel plant-based therapeutics (Agize et al., 2022). Therefore, documenting, preserving, and integrating traditional knowledge into contemporary conservation and healthcare strategies should be regarded as a critical component of the One Health approach.

To address these challenges, conservation strategies must adopt an integrated and ecosystem-based approach. This means managing biodiversity as a dynamic system where humans, culture, and environment coexist. *In-situ* conservation through protected areas, forest reserves, and plant micro reserves remains the cornerstone of maintaining wild populations (Gaur et al., 2024). Meanwhile, *ex-situ* approaches such as seed banks and botanical gardens provide complementary safeguards for rare or endangered species. Promoting the cultivation of medicinal plants through sustainable agricultural systems, including agroforestry, can further relieve harvesting pressure on wild populations while providing economic opportunities for rural communities.

Equally important is the promotion of sustainable harvesting practices to ensure ecological balance and long-term species viability (Schippmann et al., 2006). Sustainable harvest strategies should consider multiple levels from landscape and ecosystem integrity to plant population and genetic structure (van Wyk and Prinsloo, 2018). Techniques such as rotational harvesting, selective collection, and the use of non-destructive plant parts have shown promising results (Asigbaase et al., 2023; Zaman et al., 2025). For instance, studies on *Neopicrorhiza scrophulariiflora* (Pennell) D.Y.Hong (Plantaginaceae) demonstrate that low-intensity harvesting (25% removal) allows natural regeneration within a single year under suitable conditions, highlighting the potential of evidence-based management (Poudeyal et al., 2024).

Finally, traditional knowledge and community participation form the ethical and practical backbone of conservation (Gaur et al., 2024). Indigenous and local communities have long developed adaptive systems, such as sacred grove protection, seasonal harvesting taboos, and rotational land use that promote ecological harmony (Asigbaase et al., 2023). Integrating this wisdom into modern conservation frameworks not only enhances the sustainability of MAP use but also reinforces cultural identity and resilience. Ethnobotanical documentation is thus an urgent priority to preserve vanishing knowledge systems and guide future conservation planning.

The sustainable management of medicinal and aromatic plants requires a holistic vision that integrates ecology, culture, and health under the One Health paradigm. By preserving and integrating traditional knowledge with scientific innovation and community stewardship, it becomes possible to conserve biodiversity, safeguard medicinal resources, and sustain the connections between human wellbeing and the natural world. Although medicinal plants have been proposed as indicators of ecosystem health, empirical evidence directly linking phytochemical variability with ecosystem resilience remains limited. Longitudinal ecological studies integrating environmental monitoring and metabolomic analyses are still limited.

### 3.4 Regional case studies illustrating One Health applications of medicinal plants

Regional experiences provide practical examples of how medicinal plants contribute to One Health objectives. In Mali, medicinal plants such as *Combretum micranthum* G. Don (Combretaceae; kinkéliba), *Mitragyna inermis* (Willd.) Kuntze (Rubiaceae), and *Trichilia emetica* Vahl (Meliaceae) are traditionally used to manage malaria-related symptoms and pregnancy-associated health conditions, demonstrating the continued importance of ethnomedicine in human healthcare (Haidara et al., 2016). Similarly, in Ethiopia, medicinal plants including *Allium sativum* L. (Amaryllidaceae), *Dodonaea viscosa* subsp. *Angustifolia* (L.f.) J.G.West (Sapindaceae), and *Ocimum lamiifolium* Hochst. ex Benth. (Lamiaceae) are used for infectious diseases, wound healing, and pain management, reflecting the integration of medicinal plant knowledge into community healthcare systems (Gedlu et al., 2024).

From an animal health perspective, ethnoveterinary practices in many African and Asian regions utilize medicinal plants to manage parasitic infections, improve livestock productivity, and reduce dependence on conventional antimicrobials. Examples include the use of *Allium sativum* L. (Amaryllidaceae), *Zingiber officinale* Roscoe (Zingiberaceae; ginger)*,* and *Punica granatum* L. (Lythraceae; pomegranate) against gastrointestinal parasites in livestock and poultry systems (Ali et al., 2019).

The Himalayan region provides an environmental case study in which medicinal plants such as *Neopicrorhiza scrophulariiflora* (Pennell) D.Y.Hong (Plantaginaceae), *Dactylorhiza hatagirea* (D.Don) Soó (Orchidaceae), and *Nardostachys jatamansi* (D.Don) DC. (Caprifoliaceae) face increasing threats from overharvesting, habitat degradation, and climate change (Manish, 2022). These species illustrate how biodiversity conservation, sustainable resource management, and healthcare resilience are interconnected with the One Health framework. These regional examples suggest that medicinal plants can serve as links among human health, animal health, and environmental sustainability across diverse socio-ecological contexts.

### 3.5 Medicinal plants as biological and socio-ecological mediators in One Health

**FIGURE 1:**
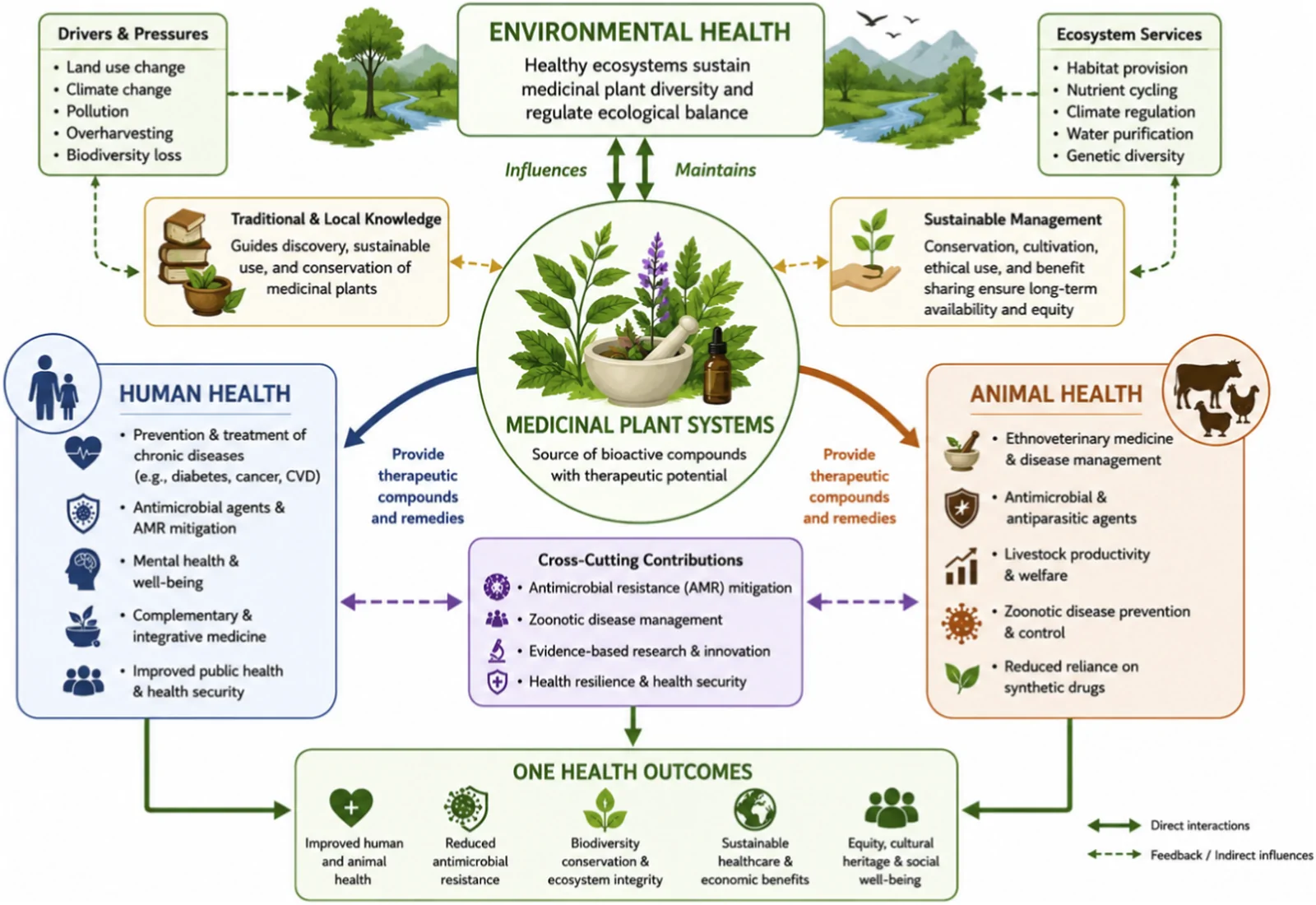
Conceptual framework illustrating medicinal plants as biological and socio-ecological mediators within the One Health continuum.

Medicinal plants function as mediators within the One Health framework because they occupy a unique position at the interface of human, animal, and environmental systems (Go et al., 2026). Unlike conventional pharmaceuticals, medicinal plants are living biological resources whose therapeutic efficacy, availability, and ecological functions are influenced by environmental conditions while simultaneously affecting human and animal health outcomes (Davis and Choisy, 2024). The interactions between environmental health, medicinal plant systems, animal health, and human health are summarized in the conceptual framework presented in Figure 1.

Environmental conditions influence medicinal plant diversity, phytochemical quality, and availability, which in turn affect human and animal health. Traditional knowledge, sustainable management, antimicrobial resistance mitigation, ethnoveterinary medicine and zoonotic disease control create feedback pathways linking ecosystem integrity, animal health, and human wellbeing.

At the environmental level, medicinal plants respond to climatic conditions, soil quality, biodiversity status, and ecosystem disturbances (Takubessi et al., 2025). Environmental stressors can alter plant growth, distribution, and phytochemical composition, thereby influencing the production of bioactive compounds. Changes in medicinal plant populations may therefore serve as indicators of ecosystem health and environmental degradation (Ogwu et al., 2025).

At the human and animal health levels, these bioactive compounds may contribute to disease prevention and treatment under specific experimental or traditional use contexts (Contreras-Pacheco et al., 2026). In livestock systems, medicinal plants support ethnoveterinary practices and may reduce dependence on conventional antimicrobials, thereby helping to mitigate antimicrobial resistance and reduce the risk of zoonotic disease transmission (Batool et al., 2025).

The interactions among these domains create a feedback system. Environmental degradation reduces medicinal plant diversity and alters phytochemical profiles, which may diminish therapeutic efficacy and limit healthcare resources available to human and animal populations (Jangpangi et al., 2025). Unsustainable harvesting driven by healthcare demands can further accelerate biodiversity loss. Traditional knowledge acts as an additional connecting component by guiding sustainable use, conservation practices, and therapeutic applications across generations (Alum, 2025).

Therefore, medicinal plants should be reviewed not merely as therapeutic resources but as biological and socio-ecological mediators that facilitate interactions between ecosystem integrity, animal health, and human wellbeing. Their conservation and sustainable management are consequently essential for strengthening resilience across the entire One Health continuum.

### 3.6 Critical synthesis, knowledge gaps and future research priorities

It is important to note that the therapeutic, veterinary, and ecological benefits discussed throughout this review vary considerably in the strength of supporting evidence. While some medicinal plants and plant-based compounds have been evaluated through pharmacological, toxicological, veterinary, and clinical studies, many reported applications remain supported primarily by traditional knowledge, observational reports, or preclinical investigations. Furthermore, differences in study design, plant preparation methods, dosage, and outcome measurements may contribute to inconsistencies among reported findings. Claims regarding efficacy, safety, and ecological benefits should be interpreted within the context of the available evidence and the limitations of current research.

Despite the growing recognition of medicinal plants within human healthcare, ethnoveterinary medicine, and biodiversity conservation, several critical knowledge gaps remain. One major limitation is the predominance of descriptive and laboratory-based studies. Although numerous medicinal plants have demonstrated promising antimicrobial, anti-inflammatory, antioxidant, and immunomodulatory activities, much of the evidence is derived from *in vitro* experiments or traditional use reports. Comparatively fewer studies have validated these findings through well-designed clinical trials (Trujillo Frede et al., 2025), veterinary field studies, or long-term ecological assessments. Consequently, the translation of medicinal plant research into evidence-based One Health interventions remain limited (Nielsen et al., 2012).

Another challenge is the lack of standardization. The phytochemical composition of medicinal plants can vary substantially according to genetic factors, geographical origin, cultivation practices, harvesting time, and environmental conditions. Such variability affects the reproducibility of biological activities and complicates the development of standardized therapeutic products (Dawoud, 2025). Establishing quality control protocols and integrating phytochemical profiling into medicinal plant research should therefore be considered a priority for future investigations.

Despite their considerable therapeutic potential, medicinal plants also present several limitations that should be considered when translating traditional knowledge into evidence-based healthcare applications. Safety considerations represent another important challenge in the application of medicinal plants. Although medicinal plants are often perceived as inherently safe because of their natural origin, several species contain bioactive compounds that may produce adverse effects, toxicity, or clinically significant herb-drug interactions (Zhang et al., 2026). Toxicity may arise from inappropriate dosing, prolonged use, contamination of heavy metals or pesticides, adulteration, or misidentification of plant species. For example, *Peganum harmala* L. (Nitrariaceae; Syrian rue) contains β-carboline alkaloids that may cause neurotoxic and hallucinogenic effects at high doses (Herraiz et al., 2010), while excessive consumption of *Glycyrrhiza glabra* L. (Fabaceae; licorice) has been associated with hypertension, hypokalemia, and cardiovascular complications (Ceccuzzi et al., 2023). In addition, certain herbal products may alter the pharmacokinetics of conventional medicines, potentially affect therapeutic efficacy or increase the risk of adverse reactions (Gamil et al., 2025). These concerns emphasize the importance of toxicological assessment, dosage standardization, quality control, and post-market safety monitoring to ensure the safe use of medicinal plants in both human and veterinary healthcare systems.

One major challenge is the lack of standardized dosage recommendations, as the concentration of bioactive compounds can vary considerably according to plant species, geographical origin, cultivation conditions, harvesting time, and extraction methods. Some medicinal plants may exhibit toxic effects, herb-drug interactions, or adverse reactions when consumed at inappropriate doses or combined with conventional medications (Busia, 2024). Bioavailability also remains a significant limitation, as many phytochemicals demonstrate poor absorption, rapid metabolism, or limited systemic distribution, which may reduce their clinical efficacy despite promising *in vitro* activity (Stielow et al., 2023).

In addition, much of the current evidence supporting medicinal plant use originates from laboratory studies and traditional knowledge systems, whereas robust clinical trials and veterinary field studies remain comparatively limited (Akram and Mahmood, 2024). Addressing these challenges through standardized formulations, pharmacokinetic investigations, toxicity assessments, and well-designed clinical studies will be essential for the safe and effective integration of medicinal plants into contemporary One Health strategies.

Translational barriers also remain a major obstacle to the broader application of medicinal plants in evidence-based healthcare, veterinary medicine, and One Health policy. A central challenge is phytochemical variability, as the concentration and composition of active compounds may differ according to plant genotype, geographical origin, cultivation conditions, harvest season, plant part used, post-harvest processing, extraction method, and storage conditions. This variability complicates reproducibility and makes it difficult to establish consistent dosage, safety, and efficacy profiles. Quality control is therefore essential and should include botanical authentication, chemical fingerprinting, quantification of marker compounds, screening for contaminants such as heavy metals, pesticides, mycotoxins, and microbial impurities, and verification against adulteration or substitution.

The regulatory approval remains challenging because medicinal plant preparations often contain multiple bioactive compounds rather than a single defined active ingredient, making pharmacokinetic evaluation, mechanism of action studies, and clinical trial design more complex. Commercialization is also limited by difficulties in large-scale cultivation, sustainable sourcing, batch-to-batch consistency, intellectual property protection, benefit-sharing with local communities, and compliance with national and international regulatory standards. Addressing these translational barriers will be essential for converting traditional and experimental medicinal plant knowledge into standardized, safe, and scalable products for human and animal health applications.

The role of medicinal plants in combating antimicrobial resistance also requires further exploration. Current evidence suggests that plant-derived compounds may disrupt microbial membranes, inhibit biofilm formation, suppress efflux pumps, and enhance the efficacy of conventional antibiotics. However, most studies remain limited to laboratory settings, and the long-term effectiveness, safety, pharmacokinetics, and ecological consequences of widespread application are not yet fully understood (Lai et al., 2022). Future research should focus on translational studies that evaluate medicinal plants as complementary strategies for antimicrobial resistance management in both human and veterinary medicine.

From an environmental perspective, the impacts of climate change on medicinal plant resources remain insufficiently understood. Environmental stressors such as rising temperatures, precipitation patterns, and habitat degradation can influence species distribution, productivity, and secondary metabolite production. These changes may directly affect the therapeutic properties of medicinal plants and indirectly influence healthcare systems that depend on them (Abbasi et al., 2023). Longitudinal studies examining the relationship between climate change, phytochemical variation, and medicinal efficacy are therefore urgently needed.

The traditional knowledge remains underrepresented in contemporary scientific research despite its central role in medicinal plant utilization and conservation. The continued erosion of indigenous and local knowledge systems threatens not only cultural heritage but also the sustainable management of medicinal plant resources. Future One Health initiatives should promote participatory research approaches that integrate traditional ecological knowledge with modern scientific methodologies.

Finally, there is a need for systems-level investigations that explicitly examine medicinal plants as biological and socio-ecological mediators linking human, animal, and environmental health. Future studies should adopt interdisciplinary frameworks combining phytochemistry, ecology, ethnobotany, veterinary sciences, public health, and conservation biology. Such approaches would provide a more comprehensive understanding of how medicinal plants contribute to ecosystem resilience, disease prevention, and sustainable healthcare within the One Health paradigm.

### 3.7 Policy integration and future perspectives within One Health systems

The growing recognition of medicinal plants within the One Health framework presents important opportunities for both policy development and interdisciplinary collaboration. Despite their widespread use in human healthcare, ethnoveterinary medicine, and biodiversity conservation, medicinal plants remain insufficiently represented in many national and international One Health strategies. Future policies should recognize medicinal plants as resources that connect human, animal, and environmental health through interconnected biological, ecological, and socio-cultural pathways.

From a public health perspective, greater efforts are needed to establish evidence-based guidelines for the safe and effective use of medicinal plants, including standardized quality control measures, phytochemical characterization, toxicity assessment, and pharmacovigilance systems. In veterinary medicine, validated ethnoveterinary practices may provide complementary approaches for reducing antimicrobial use and supporting sustainable livestock management. At the environmental level, medicinal plant conservation should be integrated into biodiversity protection strategies, ecosystem restoration programs, and climate adaptation policies.

Future One Health initiatives should promote collaboration among healthcare professionals, veterinarians, ecologists, conservation biologists, policymakers, and indigenous communities. Emphasis should be placed on preserving traditional knowledge, supporting sustainable cultivation and harvesting practices, and ensuring equitable benefit sharing associated with medicinal plant resources. Advances in phytochemistry, biotechnology, and systems biology may further enhance the translation of medicinal plant research into evidence-based applications.

Integrating medicinal plants into One Health governance frameworks may strengthen healthcare resilience, support biodiversity conservation, and contribute to sustainable development goals. Such an approach would help maximize the ecological, therapeutic, and socio-economic benefits of medicinal plants while addressing emerging challenges associated with environmental change, antimicrobial resistance, and global health security.

## 4 Conclusion

Medicinal plants occupy a unique and underexplored position at the intersection of human health, animal health, and environmental integrity. Despite their extensive use in healthcare systems worldwide, they have often been treated as passive resources rather than dynamic biological and socio-ecological components of One Health systems.

By synthesizing evidence from human medicine, ethnoveterinary practice, and environmental sciences, this review highlights the multifaceted roles of medicinal plants in supporting health, contributing to antimicrobial resistance mitigation, and linking biodiversity conservation with healthcare resilience. The findings suggest that medicinal plants may function not only as therapeutic resources but also as mediators connecting ecosystem integrity, animal health, and human wellbeing.

However, important challenges remain, including limited clinical validation, phytochemical variability, standardization issues, regulatory constraints, and threats arising from biodiversity loss, climate change, and the erosion of traditional knowledge. Addressing these challenges will require interdisciplinary research, supportive policy frameworks, sustainable resource management, and stronger integration of medicinal plants into One Health strategies.

Ultimately, recognizing medicinal plants as biological and socio-ecological mediators may contribute to more resilient, evidence-based, and sustainable approaches to global health and environmental stewardship.
