# Supplementary material for: Medicinal plants as biological and socio-ecological components of One Health
Source: Front Pharmacol. 2026 Aug 12;17:1868266. doi: 10.3389/fphar.2026.1868266 (PMC13507835; doi:10.3389/fphar.2026.1868266)
Supplement: Supplementary file 1 [file Table1.docx]

Supplementary Material

# 1 Supplementary Data

Supplementary Table 1. Representative medicinal plants, major bioactive compounds, and reported therapeutic applications in human health

| Therapeutic Category | Representative Medicinal Plant  (Major Bioactive Compound(s)) | Reported Therapeutic Application | Ref. |
| --- | --- | --- | --- |
| Antimalarial / Anti-protozoal | *Aerva lanata* | Antiplasmodial (Good activity against *Plasmodium falciparum* K1) | [4, 9, 53] |
|  | *Aframomum giganteum* | Antiplasmodial (Moderate activity against *P. falciparum* Fcbm W2) |  |
|  | *Ajuga remota* | Antiplasmodial; Antimalarial |  |
|  | *Alchornea cordifolia* | Antiplasmodial (Very Good, consistently reported) |  |
|  | *Aloe debrana* | Antimalarial |  |
|  | *Aloe pulcherrima* | Antiplasmodial |  |
|  | *Alstonia boonei* | Antimalarial |  |
|  | *Alstonia congensis* | Antiplasmodial (Very Good against *P. falciparum* K1) |  |
|  | *Amaranthus spinosus* | Antimalarial (*in vivo*) |  |
|  | *Ampelocissus africana* | Antiplasmodial |  |
|  | *Annona muricata* | Antiplasmodial (potently inhibits *P. falciparum*) |  |
|  | *Annona reticulata* | Antiplasmodial (potently inhibits *P. falciparum*) |  |
|  | *Anthocleista djalonensis* | Antiplasmodial |  |
|  | *Antiaris toxicaria* | Specific traditional use (by key informants) |  |
|  | *Artemisia annua* | Crude extract shows antiplasmodial activity |  |
|  | *Asparagus africanus* | Antimalarial (*in vivo*) |  |
|  | *Asparagus virgatus* | Antiplasmodial (Good activity against *P. falciparum* D10) |  |
|  | *Aspilia africana* | Antiplasmodial (Moderate activity against *P. falciparum* D10, K1) |  |
|  | *Aspilia pruliseta* | Antiplasmodial (Good activity against *P. falciparum* D6, W2) |  |
|  | *Asystasia gangetica* | Antiplasmodial (Moderate activity against *P. falciparum* D10) |  |
|  | *Azadirachta indica* | Antiplasmodial (Very Good activity against *P. falciparum* strains) |  |
|  | *Blighia welwitschii* | Malaria |  |
|  | *Boerhaavia erecta* | Antimalarial (*in vivo*) |  |
|  | *Bombax buonopozense* | Antimalarial (Good, 93% suppression rate) |  |
|  | *Brassica nigra* (Black Mustard) | Antimalarial (Moderate); Listed for use in pregnancy |  |
|  | *Brucea antidysenterica* | Antimalarial (*in vivo*) |  |
|  | *Brucea javanica* | Antiplasmodial (Very good against *P. falciparum* W2) |  |
|  | *Bruguiera gymnorhiza* | Antiplasmodial (Moderate activity against *P. falciparum* D10) |  |
|  | *Caesalpinia bonduc* | Antiplasmodial |  |
|  | *Caesalpinia volkensii* | Antimalarial |  |
|  | *Carica papaya* (Papaya) | Antiplasmodial; Listed for use in pregnancy |  |
|  | *Celtis integrifolia* | Antiplasmodial/Antimalarial (Very good) |  |
|  | *Chrysophyllum perpulchrum* | Antiplasmodial (Moderate activity against *P. falciparum* FCB1) |  |
|  | *Cinchona succirubra* | Antiplasmodial (Good activity against *P. falciparum* strains) |  |
|  | *Clerodendrum myricoides* | Antimalarial |  |
|  | *Clutia abyssinica* | Antimalarial (Moderate) |  |
|  | *Clutia hirsuta* | Antiplasmodial (Moderate activity against *P. falciparum* D10) |  |
|  | *Clutia robusta* | Antiplasmodial (Good activity against *P. falciparum* 3D7, DD2) |  |
|  | *Cocos nucifera* | Antimalarial |  |
|  | *Cochlospermum planchonii* | Anti-malarial (tested in a clinical trial) |  |
|  | *Combretum glutinosum* | Treatment of malaria symptoms (in pregnancy) |  |
|  | *Combretum micranthum* | Most commonly mentioned plant for malaria symptoms (in pregnancy, Mali); Antiplasmodial |  |
|  | *Croton macrostachyus* | Antimalarial (*in vivo*) |  |
|  | *Cryptolepis sanguinolenta* | Antiplasmodial (Very Good, consistently reported) |  |
|  | *Cucumis metuliferus* | Antimalarial (Moderate, 70.69% suppression rate) |  |
|  | *Dacryodes edulis* | New antimalarial hits |  |
|  | *Dichapetalum guineense* | Antiplasmodial (Moderate activity against *P. falciparum* 3D7 & K1) |  |
|  | *Dichrostachys cinerea* (Wight et Arn) | Antiplasmodial (Very Good against *P. falciparum* NF54) |  |
|  | *Dicoma anomala* | Antiplasmodial |  |
|  | *Dicoma tomentosa* | Antiplasmodial |  |
|  | *Diospyros mespiliformis* | Used for Mumps (human ailment); Antimalarial (Very good against *P. falciparum* strains) |  |
|  | *Dodonea angustifolia* | Antimalarial |  |
|  | *Eclipta prostrata* | Malaria symptoms; Used for anti-protozoan effects |  |
|  | *Entandrophragma caudatum* | Antiplasmodial (Very Good against *P. falciparum* UP1) |  |
|  | *Entandrophragma palustre* | Antiplasmodial |  |
|  | *Euphorbia hirta* | Antimalarial |  |
|  | *Ficus thonningii* | Antiplasmodial; Antimalarial |  |
|  | *Flueggea virosa* | Antiplasmodial (Very Good, consistently reported) |  |
|  | *Gardenia ternifolia* | Antimalarial |  |
|  | *Gutenbergia cordifolia* | Antiplasmodial/Antimalarial (Very good) |  |
|  | *H. suaveolens* | Antiplasmodial (Very Good against *P. falciparum* D10) |  |
|  | *Haplophyllum tuberculatum* | Antiplasmodial (Very Good against *P. falciparum* 3D7, Dd2) |  |
|  | *Harungana madagascariensis* | Anti-protozoan effects (against *Trichomonas* and *Plasmodium*); Antimalarial |  |
|  | *Harrisonia abyssinica* | Antiplasmodial (Very Good against *P. falciparum* W2, SHF4) |  |
|  | *Helchrysum cymosum* | Antiplasmodial/Antimalarial (Very good) |  |
|  | *Holarrena floribunda* | Antimalarial |  |
|  | *Hypoestes forskaolii* | Antiplasmodial (Good activity against *P. falciparum* D6, W2) |  |
|  | *Hyptis pectinata* | Antiplasmodial (Moderate activity against *P. falciparum* D10) |  |
|  | *Icacina senegalensis* | Antiplasmodial/Antimalarial (Very good); inhibits *P. falciparum* growth |  |
|  | *Khaya grandifoliola* | Antimalarial (limonoids) |  |
|  | *Kniphofia foliosa* | Antimalarial |  |
|  | *Lannea schweinfurthii* | Antiplasmodial/Antimalarial (Very good) |  |
|  | *Lippia chevalieri* | Malaria symptoms (in pregnancy); Antiplasmodial |  |
|  | *Lippia javanica* | Antiplasmodial (Good, Moderate activity against *P. falciparum* strains) |  |
|  | *Lippia kituiensis* | Moderate activity against *Plasmodium berghei* ANKA |  |
|  | *Lophira lanceolata* | Moderate activity against *Plasmodium berghei* |  |
|  | *Maerua crassifolia* | Moderate activity against *Plasmodium berghei* NK65 |  |
|  | *Maytenus senegalensis* | Antiplasmodial (Very Good, consistently reported); Antimalarial (*in vivo*, 98.1% suppression rate) |  |
|  | *Mezoneuron benthamianum* | Antiplasmodial |  |
|  | *Microglossa pyrifolia* | Antiplasmodial/Antimalarial (Very good) |  |
|  | *Mikania cordata* | Antiplasmodial (Moderate activity against *P. falciparum* K1) |  |
|  | *Millettia zechiana* | Antiplasmodial (Moderate/Good activity against *P. falciparum* strains) |  |
|  | *Mitragyna inermis* | Treatment of malaria symptoms (in pregnancy) |  |
|  | *Momordica charantia* (Bitter Melon) | Antiplasmodial; contains anti-HIV proteins MAP30 and MRK29 |  |
|  | *Morinda morindoides* | Antimalarial (Good, 74% suppression rate) |  |
|  | *Moringa oleifera* | Antiplasmodial (Moderate) |  |
|  | *Mucuna pruriens* | Antiplasmodial (Good, 71.75% suppression rate) |  |
|  | *Nauclea latifolia* | Antiplasmodial |  |
|  | *Nauclea pobeguinii* | Antiplasmodial (Inactive in tested strain) |  |
|  | *Nigella sativa* (Black Cumin) | Antimalarial; Listed for use in pregnancy |  |
|  | *Ocimum lamiifolium* | Antimalarial; Used for fibril illness; Listed for use in pregnancy |  |
|  | *Osyris quadripartita* | Effect of crude leaf extract on *Plasmodium berghei* in mice |  |
|  | *Opilia celtidifolia* | Antiplasmodial/Antimalarial (Very good) |  |
|  | *Pappea capensis* | Antiplasmodial (Very Good against *P. falciparum* NF54) |  |
|  | *Parinari curatellifolia* | Antiplasmodial (Very Good against *P. falciparum* strains) |  |
|  | *Parkia biglobosa* | Treatment of malaria symptoms/UTI (in pregnancy) |  |
|  | *Paullinia pinnata* | Malaria |  |
|  | *Phyllanthus amarus* | Antiplasmodial; Antimalarial |  |
|  | *Phyllanthus niruri* | Antimalarial |  |
|  | *Phytolacca dodecandra* | Antiplasmodial (*in vivo*); Top choice for rabies in livestock |  |
|  | *Picralima nitida* | Antiplasmodial (Good against *P. falciparum* D10) |  |
|  | *Pittosporum viridiflorum* | Antimalarial (Moderate, 54.77% suppression rate) |  |
|  | *Prunus africana* | Antiplasmodial (Moderate activity against *P. falciparum* D6, W2); Second most effective against blackleg (livestock) |  |
|  | *Pseudospondias microcarpa* | Antiplasmodial (Very Good against *P. falciparum* K1) |  |
|  | *Psiadia punctulata* | Antiplasmodial (Good activity against *P. falciparum* D10) |  |
|  | *Quassia Africana* | Antiplasmodial/Antimalarial (Very good) |  |
|  | *Rhus natalensis* | Antimalarial (Moderate, 82.7% suppression rate) |  |
|  | *Ricinus communis var. communis* | Antiplasmodial (Good activity against *P. falciparum* D10) |  |
|  | *Rubia cordifolia* | Antiplasmodial |  |
|  | *Rumex abyssinicus* | Antiplasmodial/Antimalarial (Very good); Fifth most effective against blackleg (livestock) |  |
|  | *Salacia nitida* | Antimalarial (Moderate, 71.15% suppression rate) |  |
|  | *Salvia radula* | Antiplasmodial |  |
|  | *Sarcocephalus latifolius* | Malaria symptoms (in pregnancy); UTI |  |
|  | *Sclerocarya birrea* | Antiplasmodial (Moderate activity against *P. falciparum* D6, W2) |  |
|  | *Secamone afzelii* | Antiplasmodial (Moderate activity against *P. falciparum* 3D7 & K1) |  |
|  | *Sida acuta* | Antimalarial |  |
|  | *Stachytarpheta cayennensis* | Antimalarial (Good, 78.2% suppression rate) |  |
|  | *Strychnos icaja* | Antiplasmodial (Very good against *P. falciparum* strains) |  |
|  | *Strychnos spinosa* | Antiplasmodial (Moderate activity against *P. falciparum*) |  |
|  | *Stylosanthes erecta* | Treatment of malaria symptoms (in pregnancy) |  |
|  | *Telfairia occidentalis* | Antimalarial (Good, 72.17% suppression rate) |  |
|  | *Tetrapleura tetraptera* | Antiplasmodial (Moderate) |  |
|  | *Thalia geniculata* | Antiplasmodial (Moderate activity against *P. falciparum* 3D7 & K1) |  |
|  | *Tithonia diversifolia* | Antimalarial (Good, 74.97% suppression rate) |  |
|  | *Toddalia asiatica* | Antimalarial (Moderate) |  |
|  | *Tragia benthamii* | Antimalarial |  |
|  | *Trichilia emetica* | Malaria symptoms (in pregnancy); Tiredness |  |
|  | *Tridax procumbens* | Antiplasmodial |  |
|  | *Verbascum sinaiticum* | Used to treat febrile diseases; fourth most effective against blackleg (livestock) |  |
|  | *Vernonia amygdalina* | Antiplasmodial; Antimalarial; Listed for use in pregnancy; treats intestinal parasite |  |
|  | *Vepris heterophylla* | Malaria symptoms (in pregnancy); Antimalarial |  |
|  | *Vitex doniana* | Malaria symptoms (in pregnancy) |  |
|  | *Warburgia stuhlmannii* | Antiplasmodial (Very Good against *P. falciparum* D6, W2) |  |
|  | *Ximenia americana* | Antiplasmodial (Very Good against *P. falciparum* strains); Wound treatment |  |
|  | *Zanthoxylum chalybeum* | Antiplasmodial (Very Good, consistently reported) |  |
|  | *Ziziphus cambodiana* | Antiplasmodial (Moderate activity against *P. falciparum* W2) |  |
| Antimicrobial / Anti-Infectious (Bacterial/Fungal/Viral) | *Acacia nilotica* | Antibacterial (*Staphylococcus aureus*); Colic pain (livestock) | [5, 13, 54, 55] |
|  | *Acalypha wilkensiana* | Analgesic, Antimalarial |  |
|  | *Acmella caulirhiza* | Used for loose tooth |  |
|  | *Acmella paniculata* | Antibacterial (*E. aerogenes*) |  |
|  | *Adenia lobata* | Ophthalmics |  |
|  | *Ageratum conyzoides* | Headache |  |
|  | *Allium sativum* (Garlic) | Antibacterial; Antiviral, Antifungal; ingredient with direct antibacterial activity; Listed for use in pregnancy |  |
|  | *Alnus nepalensis* | Anti-MRSA |  |
|  | *Alternanthera Sessile* | Antibacterial (*S. pyogenes, S. typhi*) |  |
|  | *Amaranthus caudatus* | Antibacterial (*S. aureus, E. coli, P. mirabilis*) |  |
|  | *Amaranthus hybridus* | Antibacterial |  |
|  | *Amaranthus viridis* | Livestock use (milk production) |  |
|  | *Aristolochia Indica* | Antifungal (*A. niger, A. flavus, A. fumigatus*) |  |
|  | *Artemisia afra* | Antibacterial; Antifungal (*Candida* spp.); Listed for use in pregnancy |  |
|  | *Azadirachta indica* (Neem) | Antimicrobial; anti-infective pharmaceuticals; reduces tick burden (livestock) |  |
|  | *Barleria prionitis* | Antibacterial (Gram Positive & Negative) |  |
| Cancer / Antitumor | *Alangium salvifolium* | Anticancer, Antitumor | [6, 7, 55, 56] |
|  | *Allium sativum* (Garlic) | Anticancer (S-allylcysteine and S-allylmercapto-L-cysteine) |  |
|  | *Anemarrhena asphodeloides* | Anticancer methods using extracts |  |
|  | *Camellia sinensis* (Green tea) | Anticancer (Catechin, gallocatechin, epigallocatechin); Evaluation of anticancer activity in colorectal cancer cell line |  |
|  | *Cedrus deodra* | Anticancer activities against breast, cervix, neuroblastoma, colon, liver, lung, mouth, ovary, and prostate cancer (lignans) |  |
|  | *Celastrus orbiculatus* | Inhibits gastric cancer stem cells |  |
|  | *Celastrus paniculatus* | Anticancer (β-dihydroagarofuranoid sesquiterpenes) |  |
|  | *Curcuma longa* (Turmeric) | Anticancer |  |
|  | *Diospyros mespiliformis* | Used for tumor |  |
|  | *Fagopyrum cymosum* | Used in lung cancer, gastric cancer, cervical cancer, sarcoma |  |
|  | *Ganoderma* spp. | Anticancer |  |
|  | *Gleditsia sinensis* | Anticancer methods employing extracts |  |
|  | *Hamelia patens* | Novel anticancer drug delivery system using leaf and flower extracts encapsulated in zeolite |  |
|  | *Lemon Verbena* (*Aloysia citrodora*) | Anti-tumor effects |  |
|  | *Madhuca longifolia* | Anti-tumor, Anti-cancer |  |
|  | *Nothapodytes nimmoniana* | Contains Camptothecin (anti-cancer alkaloid) |  |
|  | *Peganum harmala* | Anti-tumor |  |
|  | *Piper aduncum* | Protective effect against DMBA-induced breast cancer |  |
|  | *Pistacia lentiscus* | Anticancer |  |
|  | *Platycodon grandiflorum* | Anticancer agent |  |
|  | *Plumbago zeylanica* | Anticancer activities; reduces tumor growth; antiproliferative properties |  |
|  | *Rhus verniciflua* | Inhibits growth of human lymphoma cells (Polyphenolic compounds and flavonoids) |  |
|  | *Rubia cordifolia* | Anticancer properties |  |
|  | *Salvia radula* | Anticancer (compounds isolated showed little toxicity) |  |
|  | *Saraca asoca* | Anti-tumor, Anti-cancer |  |
|  | *Sauropus chinensis* | Effective in liver, prostate, breast, mammary gland, and stomach cancer (Epimanassantin A and manassantin A) |  |
|  | *Serenoa repens* | Prostate cancer treatment |  |
|  | *Solanum dulcamara* | Treating prostate cancer |  |
|  | *Sphaeranthus indicus* | Anticancer |  |
|  | *Viscum album* (European mistletoe) | Anticancer (Phenolic acid, flavonoid) |  |
| CNS / Neurological / Psychological | *Ageratum conyzoides* | Headache | [5-7, 9, 55, 57, 58] |
|  | *Alangium salvifolium* | Antiepileptic, Anticonvulsant |  |
|  | *Areca catechu* | Stimulant (Nicotinic receptor agonist) |  |
|  | *Atropa belladonna* | Depressant and euphoric (Anticholinergic) |  |
|  | *Brugmansia aurea* | Depressant and euphoric (Anticholinergic) |  |
|  | *Cannabis sativa* | Stimulant, euphoria (Cannabinoid receptor agonist) |  |
|  | *Datura stramonium* | For intelligence (Human); Listed for use in pregnancy |  |
|  | *Ecballium elaterium* | Attenuates neuroinflammation in Alzheimer’s disease animal model |  |
|  | *Lagerh. Mandragora officinalis* | Depressant and euphoric (Anticholinergic) |  |
|  | *Momordica balsamina* | CNS disorders |  |
|  | *Myrica salicifolia* | Treats Brain abscess (neqerisa) |  |
|  | *Nigella sativa* (Black Cumin) | Abdominal pain |  |
|  | *Ocimum gratissimum* | Convulsion, Headaches |  |
|  | *Papaver somniferum* | Depressant and euphoric (Morphine/heroin source) |  |
|  | *Paullinia cupana* | Stimulant (Caffeine) |  |
|  | *Pimpinella anisum* (Yansun) | Neurological problems (in mixtures) |  |
|  | *Rosmarinus officinalis* (Rosemary) | Headache |  |
|  | *Rubia cordifolia* | Treats Migraine |  |
|  | *Ruta chalepensis* | Abdominal pain; Listed for use in pregnancy |  |
|  | *Salvadora persica* (Miswak) | Anticonvulsant and Sedative effects; Ameliorates anxiety and depression |  |
|  | *Salvia divinorum* | Hallucinogen (j-opioid receptor agonist) |  |
|  | *Tragia brevipes* | Cognitive enhancement (“brain enlightenment”) (Novel use) |  |
|  | *Trigonella foenum-graecum* (Fenugreek) | Neurological problems (in mixtures); Listed for use in pregnancy |  |
|  | *Withania somnifera* | Reconstruct neuronal networks; Psychoactive |  |
| Inflammation / Pain / Musculoskeletal | *Acalypha wilkensiana* | Analgesic | [4-7, 10, 55, 57, 58] |
|  | *Aesculus hippocastanum* | Anti-inflammatory properties |  |
|  | *Akebia trifoliata* (Chocolate vine) | Anti-inflammatory (chronic inflammations) |  |
|  | *Alangium salvifolium* | Antiarthritic, Antinociceptive, Analgesic, Anti-inflammatory |  |
|  | *Blighia welwitschii* | Rheumatism, Oedema |  |
|  | *Fagopyrum cymosum* | Relieving inflammation |  |
|  | *Genista tinctoria* | Anti-inflammatory (targets TNF-a, IL-1b) |  |
|  | *Hypericum gaitii* | Anti-inflammatory |  |
|  | *Madhuca longifolia* | Anti-inflammatory |  |
|  | *Mimusops elengi* (Bakul) | Analgesic |  |
|  | *Oldenlandia affinis* | Anti-inflammatory |  |
|  | *Peganum harmala* | Antioxidant, Anti-inflammatory |  |
|  | *Piper longum* (Root extract) | Anti-inflammatory |  |
|  | *Pistacia lentiscus* | Anti-inflammatory |  |
|  | *Platycodon grandiflorum* | Anti-inflammatory agent in rheumatic arthritis |  |
|  | *Pluchea indica* | Anti-inflammatory (involvement of 5-lipooxygenase pathway) |  |
|  | *Rubia cordifolia* | Anti-inflammatory properties; rheumatism |  |
|  | *Salix alba* | Early known remedy for pain and inflammation (Salycilate derivative) |  |
|  | *Terminalia chebula* | Anti-inflammatory (targets TNF-a, MMP-2) |  |
|  | *Terminalia leiocarpa* | Livestock use (e.g., musculoskeletal disorders) |  |
| Metabolic / Endocrine | *Chrysophyllum albidum* | Hypoglycemic, Hypolipidemic | [5-8, 12, 55, 58] |
|  | *Cinnamomum verum* (Cinnamon) | Effective in Type II diabetes |  |
|  | *Coptis chinensis* | Anti-diabetic (rich source of berberine) |  |
|  | *Gymnema sylvestre* | Effective in Type II diabetes |  |
|  | *Madhuca longifolia* | Treats hypertension |  |
|  | *Mimusops elengi* (Bakul) | Hypoglycemic (flower and leaf extracts) |  |
|  | *Momordica charantia* | Anti-diabetic (charantin, vicine, polypeptide-p) |  |
|  | *Nasturtium officinale* | Obesity |  |
|  | *Salvadora persica* (Miswak) | Hypoglycemic and Hypolipidemic effects |  |
|  | *Trigonella foenum-graecum* (Fenugreek) | Effective in Type II diabetes |  |
| Cardiovascular / Circulatory | *Alangium salvifolium* | Antihypertensive | [5-8, 57, 58] |
|  | *Amomum subulatum* | Block voltage-gated calcium channels (Cardamonin) |  |
|  | *Boophone disticha* | Reduce acute mixing stress induced (Alkaloid) |  |
|  | *Commiphora mukul* | Management of anginal pain, cardiac diseases (Ayurveda) |  |
|  | *Crataegus oxycantha* | Cardiotonic, antianginal, antihypertensive, lipid-lowering effects |  |
|  | *Evodia rutaecarpa* | Endothelium dependent vasorelaxant properties; antiplatelet aggregation (Rutacarpine) |  |
|  | *Terminalia arjuna* | Cardiotonic, coronary artery disease, heart failure, hypolipidemic |  |
|  | *Uncaria rhynchophylla* | Antihypertensive, antiplatelet aggregation (Rhynchophylline and hirsutine) |  |
|  | *Withania somnifera* | Cardioprotective (restored cardiac markers, improved antioxidant status) |  |
|  | *Zanthoxylum armatum* | Cardiac disorders (folklore) |  |
| Wound Healing / Dermatological / Skin | *Alangium salvifolium* | Wound healing | [5, 6, 53, 55, 57] |
|  | *Aloe vera* (Aloe leaves) | Helped heal wounds, irritations, and scorching-related skin injuries |  |
|  | *Calotropis procera* | Treats Scabies (itch) |  |
|  | *Citrus aurantiifolia* | Treats Human Wound |  |
|  | *Cordia africana* | Treats Eczema; Fire burn |  |
|  | *Glinus lotoides* | Treats Ulcer after amputation (Lufe) |  |
|  | *Madhuca longifolia* | Skin disease (seeds) |  |
|  | *Opuntia ficus-indica* | Treats Human hemorrhoids |  |
|  | *Plantago major* | Poisoning (root powder used to bandage wounds); Haemorrhoids |  |
|  | *Rubia cordifolia* | Used for wound; skin diseases |  |
|  | *Salvadora persica* (Miswak) | Wound healing potential |  |
|  | *Smilax zeylanica* | Used for ulcers and skin infections |  |
|  | *Syzygium praecox* | Used for ulcers and skin infections |  |
|  | *Verbascum sinaiticum* | Used to treat wounds; External wounds |  |
|  | *Ximenia americana* | Wound |  |
| Gastrointestinal / Digestive / Ulcer | *Alangium salvifolium* | Laxative, Antiulcer | [5-8, 10, 53, 55, 57, 58] |
|  | *Linum usitatissimum* (Flaxseed) | Heartburn/reflux problems, Constipation/obstipation, Gastritis/burning sensation, Abdominal cramps; Listed for use in pregnancy |  |
|  | *Momordica balsamina* | Gastrointestinal problems |  |
|  | *Musa acuminata* (Banana) | Digestive |  |
|  | *Myrsine africana* | Treats Taeniasis (Internal parasite) |  |
|  | *Ocimum gratissimum* | Gastritis, Stomachache |  |
|  | *Parkia biglobosa* | Gastrointestinal |  |
|  | *Phyllanthus embellica* | Hepatic disorders |  |
|  | *Phyllanthus niruri* | Hepatic disorders |  |
|  | *Pluchea indica* | Anti-ulcer |  |
|  | *Rubia cordifolia* | Dysentery |  |
|  | *Salvadora persica* (Miswak) | Antiulcer activity |  |
|  | *Tamarindus indica* (Tamarind) | Internal parasite (livestock); Digestive issues (Mecca mixtures) |  |
|  | *Tinospora cordifolia* | Hepatic disorders |  |
|  | *Trachyspermum ammi* (Nankha) | Digestive (Mecca mixture) |  |
|  | *Trigonella foenum-graecum* (Fenugreek) | Digestive (Mecca mixtures) |  |
|  | *Vernonia amygdalina* | Treats Gastritis; Intestinal parasite |  |
| Women's / Reproductive Health | *Achyranthes aspera* | Gynecological disorder | [5, 9, 10, 53, 57, 58] |
|  | *Alangium salvifolium* | Antifertility |  |
|  | *Carica papaya* (Papaya) | Used to induce labor or reduce intensity/shorten duration of labor |  |
|  | *Cinnamomum verum* (Cinnamon) | Potentially harmful in pregnancy (risk of fetal malformation and uterine contraction); Listed for use in pregnancy |  |
|  | *Hagenia abyssinica* | Potentially harmful in pregnancy (risk of uterine contraction); Listed for use in pregnancy |  |
|  | *Jatropha curcas* | Abortion |  |
|  | *Jatropha gossypiifolia* | Dysmenorrhoea |  |
|  | *Lepidium sativum* (Rashad) | Pregnancy use; Listed for use in pregnancy |  |
|  | *Linum usitatissimum* (Flaxseed) | Most commonly used in pregnancy (22.0%); preparation, induction, or shortening of labor |  |
|  | *Madhuca longifolia* | Oxytocic, uterotonic, anti-implantation, anti-progestational, antiestrogenic, anti-menorrhagia |  |
|  | *Morus nigra* | Family planning |  |
|  | *Ocimum gratissimum* | Dysmenorrhoea, Pregnancy care |  |
|  | *Otostegia integrifolia* | Manages Rh factor incompatibility (“shotelay”) during pregnancy (Novel use) |  |
|  | *Parkia biglobosa* | Most frequently cited for Urinary Tract Infections (UTI) in pregnancy |  |
|  | *Pteleopsis suberosa* | UTI |  |
|  | *Ruta chalepensis* | Potentially harmful in pregnancy (risk of uterine contraction and emmenagogue); Listed for use in pregnancy |  |
|  | *Salvadora persica* (Miswak) | Effect on fertility (male and female mice) |  |
|  | *Saraca asoca* | Antimennorhagic, Oxytocic, Uterine tonic |  |
|  | *Sarcocephalus latifolius* | UTI (in pregnancy) |  |
|  | *Stephania abyssinica* | Gynecological disorder |  |
|  | *Trigonella foenum-graecum* (Fenugreek) | Potentially harmful in pregnancy (risk of uterine contraction and hypoglycemic); Listed for use in pregnancy |  |
| Miscellaneous Uses (Tonic, General Malaise, Detoxification) | *Ageratum houstanianum* | Human ailment (General) | [5-9, 53, 57-59] |
|  | *Cordia africana* | Most threatened multipurpose species (furniture, building, fuel) |  |
|  | *Leucas martinicensis* | General malaise |  |
|  | *Nasturtium officinale* | General body tonic |  |
|  | *Olea europaea subsp. cuspidata* | Second most threatened multipurpose species (charcoal, construction, food) |  |
|  | *Opuntia ficus-indica* | Human hemorrhoids (Top preference) |  |
|  | *Pistacia lentiscus* | Antioxidant |  |
|  | *Plumbago zeylanica* | Swelling (megerem) (FL: 93.65%) |  |
|  | *Silybum marianum* (Milk thistle) | Therapeutic potential for health challenges |  |
|  | *Trichilia emetica* | Tiredness (Mali pregnancy use) |  |
|  | *Zanthoxylum armatum* | Jaundice |  |
|  | *Zehneria scabra* | Deformed lips (Megagna) |  |

Supplementary Table 2. Representative medicinal plants, major bioactive compounds, and reported therapeutic applications in animal health

| Therapeutic Category | Representative Medicinal Plant (Scientific Name) | Major bioactive compound(s) | Reported Therapeutic Application | Ref. |
| --- | --- | --- | --- | --- |
| Antimicrobial, Antiviral, & Antiparasitic  This category includes functions related to fighting infections, viruses, bacteria, and internal or external parasites (helminths, lice, ticks, fleas). | *Acacia angustissima (Mill.) Kuntze* |  | Anthelmintic activity. | [5, 16, 27, 28, 30, 32, 35, 36, 38, 60] |
|  | *Achyranthes aspera* |  | Treats Infectious disease, culturally most valuable Ethnoveterinary Medicinal Plant (EVMP). |  |
|  | *Alstonia scholaris* | blackboard tree | Known antiparasitic property (Ayurveda). |  |
|  | *Aloe zebrina* | kgophane | Treats poultry diseases including fleas and helminths. |  |
|  | *Anemone chinensis Bunge* | A. chinensis Bunge | Screened for Minimum Inhibitory Concentration (MIC) against *C. jejuni* and *E. coli*. |  |
|  | *Anogeissus leiocarpus (DC.) Guill. & Perr.* |  | Proven effective against internal parasites (anthelmintic), highly cited species (Benin). |  |
|  | *Artemisia annua* | sweet wormwood | Known antiparasitic property (Ayurveda). |  |
|  | *Asparagus flagellaris (Kunth) Baker* |  | Suggested for investigation for parasitic diseases. |  |
|  | *Azadirachta indica (neem) A.Juss.* | Neem | Known antiparasitic property (Ayurveda), culturally most valuable EVMP. |  |
|  | *Balanites aegyptiaca (L.) Delile* |  | Used for parasitic disease (Benin). |  |
|  | *Berberis calliobotries* |  | Highest fidelity level plant species (West Pakistan). |  |
|  | *Calotropis procera (Aiton) W.T.Aiton* |  | Used for parasitic disease (Benin), commonly used for ethnoveterinary practices. |  |
|  | *Cannabis sativa L.* | Motekwane | Anthelmintic in donkeys and horses. |  |
|  | *Cassia fistula* | Bahwa | Used against intestinal worms. |  |
|  | *Centella asiatica* | Gotu kola | Known antiparasitic property (Ayurveda). |  |
|  | *Cinnamomum zeylanicum* | Cinnamon | Possesses antimicrobial properties. |  |
|  | *Cucumis myriocarpus Naudin subsp myriocarpus* |  | Used to prevent internal parasites/poisoning (exposure prophylaxis). |  |
|  | *Curcuma longa L.* | Turmeric | Known antiparasitic property (Ayurveda), traditionally used to treat intestinal parasites (in mix), culturally most valuable EVMP. |  |
|  | *Cymbopogon martinii* | palm rose | Essential oil effective against *Haemonchus contortus* and *Caenorhabditis elegans*. |  |
|  | *Cymbopogon schoenanthus* | camel grass | Essential oil effective against *Haemonchus contortus*. |  |
|  | *Euphorbia unispina N.E.Br.* |  | One of the most used species to treat viral diseases (Benin). |  |
|  | *Allium sativum L.* | Garlic | Contains active antimicrobial compounds, known antiparasitic property (Ayurveda), effective against *H. contortus* larvae (in mix). |  |
|  | *Heliotropium indicum L* |  | Used for combating worms. |  |
|  | *Justicia adhatoda* | Malabar nut | Known antiparasitic property (Ayurveda). |  |
|  | *Khaya senegalensis (Desr.) A.Juss.* |  | Widely used to treat bacterial, parasitic, and viral ailments, antitrypanosomal activity, effective against internal parasites. |  |
|  | *Mallotus philippensis* |  | Used against intestinal worms (livestock). |  |
|  | *Melia azedarach* | Shunday | Used against intestinal worm. |  |
|  | *Mentha piperita L.* | Mint, Peppermint | Essential oil effective against *Haemonchus contortus*, reduces intestinal multiple resistant bacteria, reduces *E. coli* in intestines. |  |
|  | *Mitragyna inermis (Willd.) Kuntze* |  | Suggested for investigation for parasitic diseases (Benin). |  |
|  | *Moringa oleifera* | drumstick | Known antiparasitic property (Ayurveda). |  |
|  | *Myristica fragrans Houtt.* | Nutmeg | Acts as an antimicrobial. |  |
|  | *Nicotiana plumbaginifolia Viv.* | Ran-tambakhu | Acts as a vermicide. |  |
|  | *Nigella sativa L.* | Kala jira, Kalonji | Acts as antimicrobial, antiparasite, antivirus. |  |
|  | *Otostegia integrifolia* |  | Used for ascariasis, abdominal pain, livestock infestations (lice and fleas). |  |
|  | *Phytolacca dodecandra L’Hér* |  | Preferred treatment for rabies in livestock, effective against rabies, used for anthrax. |  |
|  | *Plumbago zeylanica L* |  | Possesses antibacterial and antifungal activities. |  |
|  | *Polygonum aviculare L.* | Knotweed | Promising as an antimicrobial drug or feed (7 positive effects against pathogens). |  |
|  | *Polygonum bistorta L.* |  | Used as an antimicrobial drug or feed (18 positive effects against pathogens). |  |
|  | *Polygonum hydropiper L.* |  | Demonstrates antimicrobial properties (7 positive effects against pathogens). |  |
|  | *Polygonum minus Huds.* |  | Possesses interesting antimicrobial activities. |  |
|  | *Polygonum persicaria L.* |  | Possesses interesting antimicrobial activities, showed antifungal properties (in vitro). |  |
|  | *Pseudocedrela kotschyi* |  | Suggested for investigation for parasitic diseases. |  |
|  | *Pterocarpus erinaceus* |  | Proven effective against parasites in vitro (anthelmintic). |  |
|  | *Pueraria tuberosa (Willd.) DC.* |  | Effective against Helminthiasis, culturally most valuable EVMP. |  |
|  | *Rheum officinale Baill./palmatum L.* | Rhubarb | Associated with Antifungal, Antiviral, and Antibacterial effects. |  |
|  | *Rheum undulatum* |  | High potential for reducing the intestinal worm load in livestock. |  |
|  | *Ricinus communis* |  | Effective in combating intestinal worms. |  |
|  | *Rosemary (Rosmarinus officinalis L.)* | Rosemary | Reduces intestinal multiple resistant bacteria, improves gut microbiota. |  |
|  | *Rumex acetosa (L.)* | Sorrel | Possesses antimicrobial properties (3 positive effects against pathogens). |  |
|  | *Rumex acetosella* |  | Possesses antimicrobial properties (4 positive effects against pathogens). |  |
|  | *Rumex crispus L.* | Dock | Possesses interesting antimicrobial activities (24 positive effects against pathogens). |  |
|  | *Rumex obtusifolius L.* |  | Potential as an antibacterial drug or feed (1 positive effect against pathogens). |  |
|  | *Rumex patientia L.* |  | Possesses interesting antimicrobial activities (1 positive effect against pathogens). |  |
|  | *Senna italica* | Sebetebete | Treats gastrointestinal parasites. |  |
|  | *Smilax glabra Roxb* | S. glabra Roxb | Screened for Minimum Inhibitory Concentration (MIC) against *C. jejuni* and *E. coli*, resulted in significantly higher levels of lactic acid bacteria compared to antibiotics. |  |
|  | *Sonchus asper* |  | Combats intestinal infections. |  |
|  | *Tagetes erecta* | Mexican marigold | Effective against *H. contortus* larvae (in mix with *Allium sativum*). |  |
|  | *Tamarindus indica L.* | Chinch | Treats Internal parasite, highest FL for internal parasite. |  |
|  | *Terminalia macroptera* |  | Proven effective against parasites in vitro (anthelmintic). |  |
|  | *Thymus spp.* | Thyme | Contains active antimicrobial compounds, reduces intestinal multiple resistant bacteria. |  |
|  | *Thymus gobicus* |  | Moderate potential for reducing the intestinal worm load in livestock. |  |
|  | *Thymus vulgaris (L.)* | Thyme | Used to reduce the total numbers of aerobic and coliform bacteria in the duodenum and cecum of broiler chickens. |  |
|  | *Vaccinium vitisidaea* |  | High potential for reducing the intestinal worm load in livestock. |  |
|  | *Vachellia nilotica subsp. indica (Benth.) Kyal. & Boatwr.* |  | Effective against Enteric diseases. |  |
| Gastrointestinal & Digestive Health  This category includes functions focusing on the alimentary tract, such as treating diarrhea, constipation, bloating (tympany), and acting as carminatives or purgatives. | *Abrus precatorius L.* | Rosary pea | Treats loose motion, effective against Enteric diseases. | [5, 27, 28, 30, 32, 36, 60] |
|  | *Abutilon indicum (L.) Sweet.* | Ziar guly | Carminative, aids digestive benefits. |  |
|  | *Acacia farnesiana - (L.) Willd.* | Kikar | Antidiarrheal. |  |
|  | *Acacia leucophloea (Roxb.) Wild* | Hiwar | Treats diarrhea. |  |
|  | *Achyranthes aspera* |  | Used for treating indigestion. |  |
|  | *Ajuga bracteosa* |  | Alleviates abdominal pain, acts as a carminative, treats digestive disorders, highest Use Value (UV). |  |
|  | *Amaranthus lividus* |  | Treats diarrhea. |  |
|  | *Amaranthus viridis* |  | Used for treating indigestion. |  |
|  | *Angelica archangelica* |  | Used for indigestion. |  |
|  | *Angelica cyclocarpa* |  | Used for indigestion. |  |
|  | *Angelica glauca* |  | Employed for gastrointestinal disturbances. |  |
|  | *Anethum graveolens* |  | Used for treating indigestion. |  |
|  | *Balanites aegyptiaca (L.) Delile* |  | Treats diarrhea. |  |
|  | *Biophytum sensitivum (L.) DC.* |  | Used to stop mastication (to regularize digestion process). |  |
|  | *Bombax ceiba L.* | Silk cotton tree | Treats diarrhea, treats diarrhea during pregnancy. |  |
|  | *Brassica campestris* |  | Oral solution used for abdominal discomfort. |  |
|  | *Butea monosperma (Lamk.) Taub.* | Palas | Treats tympany, treats diarrhea. |  |
|  | *Capparis decidua* |  | Used for antidiarrheal effects, digestive benefits. |  |
|  | *Carthamus oxyacantha/C. oxycantha* |  | Used to treat digestive conditions, highest Relative Frequency of Citation (RFC)/UV. |  |
|  | *Cicer arietinum L.* | Chana | Treats Tympany. |  |
|  | *Cissus quandrangularis L.* | Haddijod, Devil’s backbone | Used for multifactorial disorder (Benin). |  |
|  | *Coix lacryma-jobi L.* | Job’s tears | Treats diarrhea. |  |
|  | *Curcuma longa L.* | Turmeric | Treats Gastrointestinal disorders. |  |
|  | *Cussonia arborea Hochst. ex A. Rich.* |  | Frequently reported for multifactorial disorders (Benin). |  |
|  | *Dicoma galpinii* |  | Treats diarrhoea, stomach pain. |  |
|  | *Eclipta prostrate (L.) L.* | Maka | Treats stomach disorder. |  |
|  | *Erythrina velutina Willd.* | Mulungu | Treats Rumen meteorism (bloating). |  |
|  | *Hibiscus cannabinus L.* | Deccan hemp | Treats constipation. |  |
|  | *Holoptelia integrifolia (Roxb.) Planch.* | Lenja | Treats blood dysentery. |  |
|  | *Kalanchoe pinnata (Lam.) Pers.* |  | Treats diarrhea. |  |
|  | *Linum usitatissimum L.* | Jawas, Linseed | Treats stomach disorders. |  |
|  | *Litsea glutinosa (Lour.) C. B. Rob.* | Bolly beech | Treats loose motion (diarrhea). |  |
|  | *Mangifera indica L.* | Amba | Treats dysentery. |  |
|  | *Melia azedarach* | Shunday | Combats stomach flatulence, carminative. |  |
|  | *Mentha longifolia L.* | Enaly | Treats abdomen pain, cures stomach disorders, treats diarrhea, highest RFC/UV. |  |
|  | *Nannorrhops ritchiana (Griff.) Aitch* | Mazari | Treats mouth and foot diseases, acts as a purgative. |  |
|  | *Nasturtium officinale R. Br.* |  | Treats constipation (human use). |  |
|  | *Neolamarckia cadamba (Roxb.) Bosser* | Burflower-tree | Treats constipation (as a laxative). |  |
|  | *Nerium oleander L.* | Gander, Oleander rose-bay | Treats digestive problems. |  |
|  | *Ocimum gratissimum L.* | Ashta | Treats Tympany (bloating). |  |
|  | *Olea europaea L.* |  | Treats diarrhea. |  |
|  | *Otostegia integrifolia* |  | Used for diarrhea. |  |
|  | *Phyllanthus reticulates Poir* | Futondi | Treats Tympany (bloating) and blood dysentery. |  |
|  | *Polygonum persicaria L.* |  | Traditional use for genitourinary diseases (unconfirmed). |  |
|  | *Ricinus communis* |  | Effective in combating stomach flatulence. |  |
|  | *Rumex crispus L.* | Dock | Supports ethnoveterinary use for skin and gastrointestinal diseases. |  |
|  | *Rumex nepalensis Spreng.* | Hoola | Used for cough, indigestion, constipation. |  |
|  | *Sapindus lauriforius Vahl.* | Getla | Treats Tympany (bloating). |  |
|  | *Saussurea lappa* |  | Treats indigestion, highest RFC. |  |
|  | *Senna italica* | Sebetebete | Treats diarrhoea, stomach pain. |  |
|  | *Skimmia laureola* |  | Employed for gastrointestinal disturbances. |  |
|  | *Soymidia febrifuga (Roxb.) A. Juss.* | Indian Redwood, Rohin | Treats dysentery. |  |
|  | *Spondias mombin L.* |  | Frequently reported for multifactorial disorders (Benin). |  |
|  | *Swertia paniculata* |  | Employed for gastrointestinal disturbances. |  |
|  | *Tamarindus indica L.* | Chinch | Treats Tympany (bloating). |  |
|  | *Thymus linearis* |  | Treats indigestion, high RFC. |  |
|  | *Viburnum grandiflorum* |  | Used for indigestion. |  |
|  | *Zea mays L.* |  | Used for indigestion, used for multifactorial disorder (Benin). |  |
| Skeletal, Injury, & Wound Healing  This category includes functions related to treating trauma, injuries, fractures, burns, wounds, and localized swelling. | *Abelmoschus esculnntus (L.) Moench.* | Ranbhendi | Cures burns and injuries. | [5, 27, 28, 30, 32] |
|  | *Abutilon indicum (L.) Sweet.* | Ziar guly | Treats skin injury. |  |
|  | *Acacia catechu Willd.* | Angrezy kikar | Cure for horn injury, acts as an anticoagulant. |  |
|  | *Aerva lanata (L.) Juss. ex Schult.* | Pillow-weed | Treats fractured bone. |  |
|  | *Agave americana L.* | Century plant | Treats inflammatory swelling of the shoulder, addresses Muscular disorders, treats Dermatological disorder. |  |
|  | *Annona squamosa L.* |  | Treats Wounds. |  |
|  | *Aosa rupestres (Gardner Weigend)* | Urtiga-branca | Treats injury. |  |
|  | *Althaea officinalis L.* |  | Used to cure burns (human use). |  |
|  | *Brassica campestris* |  | Relieves muscular pain, traditionally used as a disinfectant. |  |
|  | *Butea monosperma (Lamk.) Taub.* | Palas | Treats fractures. |  |
|  | *Butea superb Roxb. Ex wild* | Velya palas | Treats fractures. |  |
|  | *Capparis decidua* |  | Bone-healing, wound healing. |  |
|  | *Carthamus oxyacantha/C. oxycantha* |  | Enhance wound healing, highest RFC/UV. |  |
|  | *Cassia tora L.* | Tarota | Treats dog bite wounds. |  |
|  | *Cayratia trifolia (L.) Domin.* | Kambarmodi | Treats Wounds. |  |
|  | *Cissus quandrangularis L.* | Haddijod, Devil’s backbone | Treats fractured bone, effective against Skeletal disorder. |  |
|  | *Citrus aurantiifolia (Christm.) Swingle* | Lomi | Treats Wound (human use). |  |
|  | *Crinum asiaticum L.* | Seashore Lily | Treats swelling warts. |  |
|  | *Croton macrostachyus Hochst. ex Delile* |  | Used in cases of finger amputation (*lufe*). |  |
|  | *Datura Metel L.* | Dhotra | Treats Wounds. |  |
|  | *Eclipta prostrate (L.) L.* | Maka | Treats wound. |  |
|  | *Gardenia gummifera L.f.* | Dikamali | Treats maggots wound healing. |  |
|  | *Helichrysum caespititium* | phateyangaka | Very effective in healing (symbolic name). |  |
|  | *Hydrolea zeylanica (L.) Vahl* | Ceylon Hydrolea | Treats Wound. |  |
|  | *Kleinia longiflora DC* | Mosiama | Treats fracture in livestock, metaphysical fracture repair. |  |
|  | *Laggera crispate* |  | Manages minor injuries and hemorrhagic conditions (first-aid practices). |  |
|  | *Merremia gangetica (L.) Cuford.* | Gopanbhaji | Treats fractures. |  |
|  | *Nicotiana rustica L.* | Aztec tobacco | Treats maggot infested wound, used as a germicide to heal cattle wounds (in mix). |  |
|  | *Plumbago zeylanica L* |  | Preferred treatment for swelling (*megerem*), effective against swelling. |  |
|  | *Rubia cordifolia L.* | Minchirur | Used for wounds, treats bone fractures. |  |
|  | *Rumex nervosus Vahl* | Barartiire | Treats Wound, root powder used to heal wound/eye problems. |  |
|  | *Sonchus asper* |  | Enhances wound healing, treats cutaneous tumors. |  |
|  | *Sphaeranthus indicus L.* | East Indian Globe Thistle | Treats Wound. |  |
|  | *Terminalia cuneata Roth.* | Arjun | Treats Wounds. |  |
|  | *Tocoyena formosa* |  | Helps in restoring fractured/broken bone. |  |
|  | *Verbascum sinaiticum Benth* |  | Treats wounds, treats burns, external wounds. |  |
|  | *Viscum angulatum Heyne ex DC.* | Khale ajan | Treats fractures. |  |
|  | *Ximenia americana L.* |  | Treats Wound (Benin). |  |
| Reproductive Health  This category includes functions related to parturition, lactation, mastitis, and fertility issues. | *Amaranthus spinosus L.* |  | Treats disorders in milk production, prescribed for delay in parturition, treats retention of milk. | [5, 27, 28, 30, 32, 36] |
|  | *Amaranthus viridis* |  | Enhancing lactation in cattle. |  |
|  | *Anethum graveolens* |  | Enhancing lactation in cattle. |  |
|  | *Bauhinia cheilantha (Bong.) Steud* | Mororó | Treats inflammation of the mammary glands. |  |
|  | *Boerhavia diffusa* | Red Spiderling | Treats post-parturition bleeding, higher use value (UV). |  |
|  | *Chenopodium album L.* | Goosefoot | Treats poor lactation. |  |
|  | *Coriandrum sativum L.* | Coriander | Treats Mastitis. |  |
|  | *Crotalaria quinquefolia L.* | Five Leaf Rattlepod | Treats sudden decrease in milk production. |  |
|  | *Cuscuta reflexa Roxb.* | Amervel | Used for abortion. |  |
|  | *Convolvulus microphyllus* | Shankhapushpi | Treats reproductive disorders. |  |
|  | *Corchorus capsularis L.* | Jute plant | Treats reproductive disorders. |  |
|  | *Dicoma galpinii* |  | Treats uterine cleansing after abortion. |  |
|  | *Dipsacus inermis* |  | Used for post-delivery treatment. |  |
|  | *Gloriosa superba L.* |  | Treats Reproductive health disorders. |  |
|  | *Grewia optiva* |  | Used for easy delivery (livestock). |  |
|  | *Grona triflora (L.) H.Ohashi & K.Ohashi* | Three-flower tick-trefoil | Treats delayed onset of estrous cycle. |  |
|  | *Ixora brachiata Roxb.* | Lokhandi | Acts as a galactagogue (enhances milk production). |  |
|  | *Linum usitatissimum L.* | Jawas, Linseed | Treats abortion. |  |
|  | *Miliusa tomentosa (Roxb.) Sinclair* | Samoka | Acts as a galactagogue (enhances milk production). |  |
|  | *Mimosa pudica L.* | Laajvanti | Treats reproductive disorders. |  |
|  | *Nerium oleander L.* | Gander, Oleander rose-bay | Treats Mastitis. |  |
|  | *Nigella sativa L.* | Kala jira, Kalonji | Treats Mastitis, used in livestock reproduction. |  |
|  | *Otostegia integrifolia* |  | Manages Rh factor complications during pregnancy. |  |
|  | *Rumex acetosa (L.)* | Sorrel | Used for post-delivery treatment. |  |
|  | *Rumex nepalensis Spreng.* | Hoola | Expelling retained placenta. |  |
|  | *Schleichera oleosa (Lour.) Oken* | Kosum | Acts as a galactagogue (enhances milk production). |  |
|  | *Senna italica* | Sebetebete | Treats retained placenta, abortions. |  |
|  | *Sonchus asper* |  | Promotes milk production. |  |
|  | *Soymidia febrifuga (Roxb.) A. Juss.* | Indian Redwood, Rohin | Treats retention of placenta, acts as a galactagogue. |  |
|  | *Taraxacum laevigatum* |  | Used for post-delivery treatment. |  |
|  | *Withania somnifera* | Modikasope | Used in the treatment of mastitis in cattle. |  |
| Metabolic, General Health, & Performance  This category includes functions related to improving overall vitality, acting as tonics, reducing fever, supporting internal organs, or enhancing performance (e.g., body weight gain (BWG), feed conversion ratio (FCR)). | *Abelmoschus moschatus Medik.* | Musk okra | Used for poor health (improving condition). | [5, 28, 30, 32, 35, 36] |
|  | *Aloe vera* |  | Effective in improving growth performance (BWG) in broilers. |  |
|  | *Aloe zebrina* | kgophane | Supports general health, treats *gala* (general malaise). |  |
|  | *Angelica cyclocarpa* |  | Acts as a tonic. |  |
|  | *Angelica glauca* |  | Employed for fever, cold, and shivering. |  |
|  | *Aralia cachemiriana* |  | Acts as a tonic. |  |
|  | *Berberis lyceum* |  | Acts as a tonic. |  |
|  | *Bistorta affinis* | B. affinis | Acts as a tonic. |  |
|  | *Bistorta amplexi-caulis* |  | Acts as a tonic. |  |
|  | *Brassica campestris* |  | Mustard oil mix used as a tonic. |  |
|  | *Cadaba aphylla (Thunb.) Wild* | Monnamontsho | Blood cleansing in cattle. |  |
|  | *Caralluma edulis* |  | Has a lower use value (UV). |  |
|  | *Caralluma tuberculata* |  | Commonly used in ethnoveterinary practices. |  |
|  | *Carthamus oxyacantha/C. oxycantha* |  | Alleviates weakness, highest RFC/UV. |  |
|  | *Centella asiatica* | Gotu kola | Higher use value (UV). |  |
|  | *Cinnamon (Cinnamomum zeylanicum)* | Cinnamon | Effective in improving growth performance, improving gut microbiota in healthy chickens. |  |
|  | *Coriandrum sativum L.* | Coriander | Antioxidant, antidiabetic, hepatoprotective. |  |
|  | *Curcuma longa L.* | Turmeric | Used in ethnoveterinary medicine. |  |
|  | *Curcumin* | Curcumin | Positive effect on poultry health. |  |
|  | *Dicoma galpinii* |  | Treats gallsickness, blood cleansing. |  |
|  | *Fagopyrum esculentum Moench* | Buckwheat | Potential antioxidative, and metabolic modifying herb, improves intake and nitrogen conversion (broiler, dairy cows), Antihyperlipidemic effects. |  |
|  | *Fagopyrum tataricum (L.) Gaertn.* |  | Potential antioxidative, and metabolic modifying herb. |  |
|  | *Fenugreek* | Fenugreek | Effective in improving growth performance, improving gut microbiota in healthy chickens, effective in reducing mortality. |  |
|  | *Garlic (Allium sativum L.)* | Garlic | Used as a minor ingredient in polyherbal remedies. |  |
|  | *Geranium wallichianum* |  | Acts as a tonic. |  |
|  | *Helianthus annus* |  | Acts as a tonic. |  |
|  | *Lemon Verbena* |  | Acts as an antioxidant, has anti-tumor effects. |  |
|  | *Lemon* | Lemon | Effective in improving growth performance, improving gut microbiota in healthy chickens. |  |
|  | *Lespedeza cuneata (Dum.Cours.) G. Don* | Chinese bushclover | Used for methane reduction in rumen. |  |
|  | *Licorice* | Licorice | Positive effect on poultry health. |  |
|  | *Linum usitatissimum L.* | Jawas, Linseed | Acts as a diuretic. |  |
|  | *Lotus corniculatus L.* | bird’s-foot trefoil | Effective in improving growth performance (BWG) in broilers, used for methane reduction in rumen. |  |
|  | *Madhuca longifolia (Koen.) Machbr.* | Moha | Treats Fever. |  |
|  | *Medicago denticulata Willd.* | Pishtari | Used as a tonic. |  |
|  | *Mentha longifolia L.* | Enaly | Acts as a diuretic, treats weakness. |  |
|  | *Mentha piperita L.* | Mint, Peppermint | Effective in promoting growth performance (BWG, FCR), reduces mortality rate, improves gut microbiota, acts as an anti-oxidant. |  |
|  | *Morus nigra* |  | Higher use value (UV). |  |
|  | *Nasturtium officinale R. Br.* |  | Higher use value (UV), acts as a diuretic, treats obesity. |  |
|  | *Ocimum lamiifolium Hochst. ex Benth* |  | Treats Fibril illness, possesses antioxidant activity. |  |
|  | *Oregano (Origanum vulgare)* | Oregano | Effective in improving growth performance, improves gut microbiota. |  |
|  | *Peganum harmala* |  | Acts as an antioxidant, anti-bacterial, anti-fungal. |  |
|  | *Polygonum aviculare L.* | Knotweed | Improves performance and affects ruminal biotransformation in sheep, antioxidative (4 positive effects). |  |
|  | *Polygonum bistorta L.* |  | Improves performance in broiler, mitigates methane emissions in ruminants. |  |
|  | *Pueraria tuberosa (Willd.) DC.* |  | Treats General weakness, treats Kidney problems. |  |
|  | *Pulicaria gnaphalodes* |  | Produced similar BWG as antibiotics, produced higher *Lactobacillus*. |  |
|  | *Rheum officinale Baill./palmatum L.* | Rhubarb | Associated with Hepatoprotective and Antihyperlipidemic effects. |  |
|  | *Rheum undulatum* |  | High potential for increasing productivity. |  |
|  | *Rosemary (Rosmarinus officinalis L.)* | Rosemary | Effective in promoting growth performance, reduces mortality rate, improves gut microbiota. |  |
|  | *Rumex hastatus* |  | Higher use value (UV). |  |
|  | *Rumex obtusifolius L.* | Dock | Improves ruminal biotransformation, prevents bloating in cows. |  |
|  | *Sageretia thea* |  | Higher use value (UV). |  |
|  | *Saussurea lappa* |  | Acts as a tonic. |  |
|  | *Senna italica* | Sebetebete | Treats *gala* (general malaise/bile reflux), supports general health, blood cleansing. |  |
|  | *Sesame* | Sesame | Effective in improving growth performance (BWG) in broilers. |  |
|  | *Skimmia laureola* |  | Employed for fever, cold, and shivering. |  |
|  | *Sonchus asper* |  | Reduces fever. |  |
|  | *Swertia paniculata* |  | Employed for fever, cold, and shivering. |  |
|  | *Thyme (Thymus spp.)* | Thyme | Effective in promoting growth performance, improves FCR, reduces mortality, improves gut microbiota. |  |
|  | *Thymus gobicus* |  | Moderate potential for increasing productivity. |  |
|  | *Tinospora cordifolia* | Guduchi | Used as an eco-friendly feed supplement and growth promoter (poultry). |  |
|  | *Tragia involucrata L.* |  | Treats Fever and related problems. |  |
|  | *Vaccinium vitisidaea* |  | High potential for increasing productivity. |  |
|  | *Verbascum sinaiticum Benth* |  | Treats febrile diseases. |  |
|  | *Withania somnifera* | Modikasope | Used for blood cleansing. |  |
| Anti-inflammatory  This category includes functions specifically targeting inflammation, pain (analgesic/antinociceptive), and related conditions like rheumatism. | *Agave americana L.* | Century plant | Treats inflammatory swelling of the shoulder. | [5, 27, 28, 30, 32, 36] |
|  | *Ajuga bracteosa* |  | Acts as an antipyretic (anti-inflammatory). |  |
|  | *Bauhinia acuminata L.* | White Bauhinia | Treats inflammatory swelling of the shoulder. |  |
|  | *Bauhinia cheilantha (Bong.) Steud* | Mororó | Treats general inflammation. |  |
|  | *Cadaba aphylla (Thunb.) Wild* | Monnamontsho | Treats pain in cattle. |  |
|  | *Calotropis gigantea (L.) W.T.Aiton* | Ruee | Treats Rheumatic disorder. |  |
|  | *Causonis trifolia (L.) Mabb. and J.Wen* |  | Treats Swelling of the body due to cold (*Jol-sannipat*). |  |
|  | *Coriandrum sativum L.* | Coriander | Anti-inflammatory. |  |
|  | *Fagopyrum esculentum Moench* | Buckwheat | Potential anti-inflammatory herb (2 positive effects). |  |
|  | *Fagopyrum tataricum (L.) Gaertn.* |  | Potential anti-inflammatory herb (1 positive effect). |  |
|  | *Hellenia speciosa (J.Koenig) S.R.Dutta* | Crepe Ginger | Treats Rheumatoid arthritis. |  |
|  | *Heliotropium indicum L* |  | Used for combating inflammation. |  |
|  | *Mentha longifolia L.* | Enaly | Recognized for its analgesic properties. |  |
|  | *Plumbago zeylanica L* |  | Possesses anti-inflammatory activities. |  |
|  | *Polygonum aviculare L.* | Knotweed | Promising as an anti-inflammatory drug or feed (50 positive effects). |  |
|  | *Polygonum bistorta L.* |  | Anti-inflammatory drug or feed (10 positive effects). |  |
|  | *Polygonum minus Huds.* |  | Possesses interesting anti-inflammatory activities (8 positive effects). |  |
|  | *Polygonum persicaria L.* |  | Possesses interesting anti-inflammatory activities (1 positive effect). |  |
|  | *Rheum officinale Baill./palmatum L.* | Rhubarb | Associated with Anti-inflammatory effects (3 positive effects). |  |
|  | *Rubia cordifolia L.* | Minchirur | Studied for anti-inflammatory properties. |  |
|  | *Rumex acetosa (L.)* | Sorrel | Possesses anti-inflammatory properties (1 positive effect). |  |
|  | *Rumex acetosella* |  | Possesses anti-inflammatory properties (4 positive effects). |  |
|  | *Rumex crispus L.* | Dock | Possesses interesting anti-inflammatory activities (31 positive effects). |  |
|  | *Rumex patientia L.* |  | Possesses interesting anti-inflammatory activities (4 positive effects). |  |
|  | *Withania somnifera* | Modikasope | Used for pain (in combination). |  |
| Specific Ailments & Toxins  This category addresses highly specific or systemic conditions, such as snakebite, rabies, cancer, foot-and-mouth disease (FMD), hemorrhoids, or urinary tract issues. | *Aegle marmelos (L.) Corr.* | Bel | Treats snakebite. | [5, 28, 30, 32, 36, 38, 60] |
|  | *Ailanthus excels Roxb.* | Maharukh | Treats FMD (Foot and Mouth Disease). |  |
|  | *Aristolochia indica L.* |  | Treats Poisonous effect. |  |
|  | *Artemisia vulgaris L.* |  | Used for hepatitis (human use). |  |
|  | *Bauhinia racemosa Lamk* | Apta | Treats scorpion bite. |  |
|  | *Cadaba spinosa (L.) K. Schum.* | Indian caper | Treats urinary tract infection with slight fever. |  |
|  | *Calotropis gigantea (L.) W.T.Aiton* | Ruee | Used for snakebite and dogbite (in mix). |  |
|  | *Capparis tomentosa* |  | Alleviates toothaches. |  |
|  | *Catharanthus roseus* | rose periwinkle | Chemical derivatives (Vinblastine, Vincristine) used for Cancer. |  |
|  | *Causonis trifolia (L.) Mabb. and J.Wen* |  | Effective against Poisonous effect. |  |
|  | *Cinchona ledgeriana* | cinchona | Chemical derivative (Quinine) used for Malaria. |  |
|  | *Cissus petiolata Hook.f* |  | Third preferred remedy for rabies in livestock, treats snake bite. |  |
|  | *Coccinia grandis (L.) Voigt* |  | Treats Ophthalmic disorder. |  |
|  | *Coffea arabica* | Arabian coffee | Chemical derivative (Caffeine) used for Fatigue, drowsiness, and migraine. |  |
|  | *Colchicum autumnale* | autumn | Chemical derivative (Colchicine) used for Gout. |  |
|  | *Commelina benghalensis L.* | Kena | Treats eye problem. |  |
|  | *Cotula anthemoides L.* | Buttonweed | Treats watering of eyes. |  |
|  | *Croton macrostachyus Hochst. ex Delile* |  | Used for rabies in livestock (ranked 4th preference). |  |
|  | *Cucumis ficifolius A.Rich* |  | Highly preferred for treating human hemorrhoids. |  |
|  | *Datura stramonium L.* |  | Treats Tinea capitis (*Quaqucha*). |  |
|  | *Ecballium elaterium* |  | Useful in Alzheimer’s disease (human use). |  |
|  | *Eheatia laevis Roxb.* | Datrangi | Used for dental treatment. |  |
|  | *Euphorbia abyssinica J.F.Gmel* |  | Treats Hemorrhoid (in mix with other latex). |  |
|  | *Euphorbia tirucalli L* |  | Treats human hemorrhoids (in mix with other latex). |  |
|  | *Ficus bengalensis L.* | Wad | Used for dental treatment. |  |
|  | *Juniperus procera* |  | Alleviates toothaches. |  |
|  | *Leonotis nepetifolia* |  | Treats anthrax. |  |
|  | *Limonia acidissima L.* | Kawath | Treats haemorrhagic septicaemia. |  |
|  | *Marsdenia rubicunda (K.Schum.) N.E.Br* |  | Used for rabies in livestock (ranked 5th preference). |  |
|  | *Myrica salicifolia Hochst. ex A.Rich* |  | Treats Brain abscess (*neqerisa*), high Fidelity Level (FL). |  |
|  | *Nannorrhops ritchiana (Griff.) Aitch* | Mazari | Treats mouth and foot diseases. |  |
|  | *Opuntia ficus-indica (L.) Mill* | Qulqual, Beles | Most preferred treatment for human hemorrhoids. |  |
|  | *Phytolacca dodecandra L’Hér* |  | Preferred treatment for rabies in livestock (Rank 1), used for jaundice, eye infections, anthrax. |  |
|  | *Primula denticulata* |  | Primarily used for treating dysuria and frequent urination. |  |
|  | *Rumex nepalensis Spreng.* | Hoola | Primarily used for treating dysuria and frequent urination. |  |
|  | *Rumex nervosus Vahl* | Barartiire | Root powder used to treat eye problems. |  |
|  | *Semicarpus anacardium L.f. (Bibba)* | Bibba | Used for dental treatment. |  |
|  | *Terminalia leiocarpa* |  | Treats eye diseases (ophthalmic applications). |  |
|  | *Tragia brevipes* |  | Used for brain enlightenment (cognitive enhancement). |  |
|  | *Tragia involucrata L.* |  | Used for Magico-religious cure. |  |
|  | *Verbascum sinaiticum Benth* |  | Second preference for rabies in livestock (Rank 2), treats snake bites, treats Anthrax, tonsillitis, toothache. |  |
|  | *Wattakaka volubilis (L.f.) Stapf.* |  | Treats Urinary disorders, culturally most valuable EVMP. |  |
|  | *Xanthium strumarium L.* | Rough cocklebur | Treats retention of urine. |  |
|  | *Ximenia americana L.* |  | Used for viral disease (Benin). |  |
| Ectoparasiticide  This category includes functions related to external pest control (e.g., lice, ticks, flies, mites). | *Abutilon indicum (L.) Sweet.* | Ziar guly | Anti-lice. | [28, 30, 32, 36, 60, 61] |
|  | *Annona squamosa L.* |  | Treats Ectoparasite (specifically listed for ectoparasite removal in India). |  |
|  | *Chloroxylon swietenia DC.* | Bhira | Used for fumigation against flies and insects. |  |
|  | *Cistus ladanifer L.* |  | Mentioned for ectoparasiticide potential. |  |
|  | *Cistus populifolius* |  | Traditional use as ectoparasiticide (first noted here). |  |
|  | *Cleistanthus collinius Roxb.* | Garadi | Used as an insecticide. |  |
|  | *Daphne gnidium L.* |  | High potential to be developed as ectoparasiticidal veterinary medicine, treats ticks, fleas, and mosquitoes/flies. |  |
|  | *Euphorbia ligularia Roxb.* | Sambar | Used as an insecticide. |  |
|  | *Juglans regia L.* | Walnut | High potential to be developed as ectoparasiticidal veterinary medicine, used against ticks, mites, and mosquitoes/flies. |  |
|  | *Lagerstroemia microcarpa Wt.* |  | Used for ticks and lice prevention. |  |
|  | *Melia azedarach* | Shunday | Antilice. |  |
|  | *Nicotiana tabacum L.* | Tobacco | Mentioned for ectoparasiticide potential, efficacy as a biocontrol agent against cattle ticks. |  |
|  | *Phytolacca dodecandra L’Hér* |  | Used for ectoparasites. |  |
|  | *Plantago lanceolata* |  | Traditional use as ectoparasiticide (first noted here). |  |
|  | *Polygonum hydropiper L.* |  | Demonstrates insecticidal properties. |  |
|  | *Ricinus communis* |  | Remedy for ear parasites (moth infestations). |  |
|  | *Ruta graveolens* | Rue | High potential to be developed as ectoparasiticidal veterinary medicine, treats ticks, fleas, and mosquitoes/flies. |  |
| Multifunctional/Research Polygonaceae  This category contains Polygonaceae species for which research data (often in *in vitro* or lab animals) detailing a wide array of biological effects (cytotoxic, hepatoprotective, etc.) was highlighted, or those 36 species with limited specific traditional use citations. | *36 Polygonaceae Species* | (Various Knotweed, Dock, Sorrel species) | Diverse biological effects including Cytotoxic, Antiviral, Antibacterial, Prebiotic, Antifungal, Hepatoprotective, Anti-inflammatory, Antihyperlipidemic, Antispasmodic, Laxative effects, and antibiotic modulatory effects. | [36] |
|  | *Fallopia aubertii (L. Henry) Holub* |  | Associated with various positive effects. |  |
|  | *Fallopia convolvulus (L.) A. Löve* |  | Associated with various positive effects, improves performance and intake (in rats). |  |
|  | *Fallopia dumetorum (L.) Holub* |  | Associated with various positive effects. |  |
|  | *Oxyria digyna (L.) Hill* |  | Associated with various positive effects. |  |
|  | *Polygonum alpinum All.* |  | Associated with various positive effects. |  |
|  | *Polygonum amphibium L.* |  | Associated with various positive effects. |  |
|  | *Polygonum arenastrum Boreau* |  | Associated with various positive effects. |  |
|  | *Polygonum capitatum D.Don* |  | Associated with various positive effects. |  |
|  | *Polygonum lapathifolium L.* |  | Associated with various positive effects. |  |
|  | *Polygonum mite Schrank* |  | Associated with various positive effects. |  |
|  | *Polygonum nepalense Meisn.* |  | Associated with various positive effects. |  |
|  | *Polygonum orientale L.* |  | Associated with various positive effects. |  |
|  | *Polygonum perfoliatum L.* |  | Associated with various positive effects. |  |
|  | *Polygonum polystachyum Meisn.* |  | Associated with various positive effects. |  |
|  | *Polygonum rurivagum Boreau* |  | Associated with various positive effects. |  |
|  | *Polygonum viviparum L.* |  | Associated with various positive effects. |  |
|  | *Reynoutria japonica Houtt.* |  | Associated with various positive effects. |  |
|  | *Reynoutria sachalinensis (F. Schmidt) Nakai* |  | Associated with various positive effects. |  |
|  | *Reynoutria x bohemica Chrtek & Chrtkova* |  | Associated with various positive effects. |  |
|  | *Rheum rhabarbarum L.* |  | Associated with various positive effects. |  |
|  | *Rumex alpestris Jacq.* |  | Associated with various positive effects. |  |
|  | *Rumex aquaticus L.* |  | Associated with various positive effects. |  |
|  | *Rumex conglomeratus Murray* |  | Associated with various positive effects. |  |
|  | *Rumex hydrolapathum Huds.* |  | Associated with various positive effects. |  |
|  | *Rumex longifolius DC.* |  | Associated with various positive effects. |  |
|  | *Rumex maritimus L.* |  | Associated with various positive effects. |  |
|  | *Rumex nebroides Campd.* |  | Associated with various positive effects. |  |
|  | *Rumex nivalis Hegetschw.* |  | Associated with various positive effects. |  |
|  | *Rumex palustris Sm.* |  | Associated with various positive effects. |  |
|  | *Rumex pulcher (L.)* |  | Associated with various positive effects. |  |
|  | *Rumex sanguineus L.* |  | Associated with various positive effects. |  |
|  | *Rumex scutatus L.* |  | Associated with various positive effects. |  |
|  | *Rumex sp.* |  | Associated with various positive effects. |  |
|  | *Rumex thasiflorus Fingerh.* |  | Associated with various positive effects. |  |
|  | *Rumex x pratensis Mert. & W.W.J. Koch* |  | Associated with various positive effects. |  |
| Research/ New Use Reports  This category lists species whose primary mention in the sources was either as a reported *new use* in a specific geographical area, or were part of a study focusing on plant compounds for performance improvement/ pathogen defense where a clear overarching traditional category was not the most appropriate fit. | *Abutilon hirtum (Lam.) Sweet* |  | Use reported as exclusively new for India. | [30, 32] |
|  | *Aerva javanica (Burm.f.) Juss. ex Schult.* |  | Use reported as exclusively new for India. |  |
|  | *Albizia procera (Roxb.) Benth.* |  | Use reported as exclusively new for India. |  |
|  | *Acrotome inflata* |  | Reported for the first time for ethnoveterinary medicine in South Africa. |  |
|  | *Adiantum philippense subsp. philippense* |  | Reported first time as EVMP from India (Pteridophyte). |  |
|  | *Cajanus goensis Dalzell* |  | Use reported as exclusively new for India. |  |
|  | *Caladium bicolor (Aiton) Vent.* |  | Use reported as exclusively new for India. |  |
|  | *Breynia vitis-idaea (Burm.f.) C.E.C.Fisch.* |  | Use reported as exclusively new for India. |  |
|  | *Coleus strobilifer (Roxb.) A.J.Paton* |  | Use reported as exclusively new for India. |  |
|  | *Cotula anthemoides L.* | Buttonweed | Use reported as exclusively new for India. |  |
|  | *Croton persimilis Müll.Arg.* |  | Use reported as exclusively new for India. |  |
|  | *Euphorbia serpens* |  | Reported for the first time for ethnoveterinary medicine in South Africa. |  |
|  | *Eulophia explanata Lindl.* |  | Use reported as exclusively new for India. |  |
|  | *Grona triflora (L.) H.Ohashi & K.Ohashi* | Three-flower tick-trefoil | Use reported as exclusively new for India. |  |
|  | *Ipomoea cairica (L.) Sweet* |  | Use reported as exclusively new for India. |  |
|  | *Ipomoea obscura (L.) Ker Gawl.* |  | Use reported as exclusively new for India. |  |
|  | *Jatropha nana Dalzell & A.Gibson* |  | Use reported as exclusively new for India. |  |
|  | *Ludwigia adscendens (L.) H.Hara* |  | Use reported as exclusively new for India. |  |
|  | *Lygodium flexuosum (L.) Sw.* |  | Root used to treat fever of goat (new use for India). |  |
|  | *Phyllodium pulchellum (L.) Desv.* |  | Use reported as exclusively new for India. |  |
|  | *Phoenix acaulis Roxb.* |  | Use reported as exclusively new for India. |  |
|  | *Piper cubeba L.f.* |  | Used as a minor ingredient in polyherbal remedies. |  |
|  | *Piper longum L.* | Long pepper | Used as a minor ingredient in polyherbal remedies. |  |
|  | *Piper nigrum L.* | Black pepper | Most preferred ingredient/highest scoring minor ingredient in polyherbal preparations. |  |
|  | *Rotheca serrata (L.) Steane & Mabb.* |  | Use reported as exclusively new for India. |  |
|  | *Seseli diffusum (Roxb. ex Sm.) Santapau & Wagh* |  | Use reported as exclusively new for India. |  |
|  | *Tacca leontopetaloides (L.) Kuntze* |  | Use reported as exclusively new for India. |  |
|  | *Thesium spp.* |  | Single-plant remedy with multiple indications, reported for the first time for ethnoveterinary medicine in South Africa. |  |
|  | *Uraria lagopoides (L.) DC.* |  | Use reported as exclusively new for India. |  |
|  | *Zingiber officinale Roscoe* | Ginger | Used as a minor ingredient in polyherbal remedies. |  |
|  | *Zingiber zerumbet (L.) Roscoe ex Sm.* |  | Use reported as exclusively new for India. |  |
| Other Herbs Mentioned Generally  This table lists general-use herbs whose specific detailed function in the sources was limited, or they were primarily mentioned as commonly used ingredients without a unique therapeutic use being specifically elaborated. | *Acacia nilotica* |  | Used in ethnoveterinary medicine. | [5, 60] |
|  | *Carissa opaca* |  | Used in ethnoveterinary medicine. |  |
|  | *Detarium microcarpum Guill. & Perr.* |  | Highly cited plant species (Benin). |  |
|  | *Parkia biglobosa (Jacq.) R.Br. ex Benth.* |  | Highly cited plant species (Benin). |  |
|  | *Sorghum bicolor (L.) Moench* |  | Highly cited plant species (Benin). |  |
|  | *Vitellaria paradoxa C.F.Gaertn.* |  | Listed (Benin). |  |
